# Nitrate-driven anaerobic oxidation of ethane and butane by bacteria

**DOI:** 10.1093/ismejo/wrad011

**Published:** 2024-01-10

**Authors:** Mengxiong Wu, Jie Li, Chun-Yu Lai, Andy O Leu, Shengjie Sun, Rui Gu, Dirk V Erler, Lian Liu, Lin Li, Gene W Tyson, Zhiguo Yuan, Simon J McIlroy, Jianhua Guo

**Affiliations:** Australian Centre for Water and Environmental Biotechnology, Faculty of Engineering, Architecture and Information Technology, The University of Queensland, St Lucia, Queensland 4072, Australia; Australian Centre for Water and Environmental Biotechnology, Faculty of Engineering, Architecture and Information Technology, The University of Queensland, St Lucia, Queensland 4072, Australia; Australian Centre for Water and Environmental Biotechnology, Faculty of Engineering, Architecture and Information Technology, The University of Queensland, St Lucia, Queensland 4072, Australia; College of Environmental and Resource Science, Zhejiang University, Hangzhou 310058, China; Centre for Microbiome Research, School of Biomedical Sciences, Translational Research Institute, Queensland University of Technology (QUT), Woolloongabba, Queensland, Australia; Computational Science Program, The University of Texas at El Paso, El Paso, TX, United States; Australian Centre for Water and Environmental Biotechnology, Faculty of Engineering, Architecture and Information Technology, The University of Queensland, St Lucia, Queensland 4072, Australia; Faculty of Science and Engineering, Southern Cross University, Lismore, New South Wales, Australia; Metabolomics Australia (Queensland Node), Australian Institute for Bioengineering and Nanotechnology, The University of Queensland, St Lucia, Queensland 4072, Australia; Department of Physics, University of Texas at El Paso, El Paso, TX, United States; Centre for Microbiome Research, School of Biomedical Sciences, Translational Research Institute, Queensland University of Technology (QUT), Woolloongabba, Queensland, Australia; Australian Centre for Water and Environmental Biotechnology, Faculty of Engineering, Architecture and Information Technology, The University of Queensland, St Lucia, Queensland 4072, Australia; School of Energy and Environment, City University of Hong Kong, Hong Kong SAR, China; Centre for Microbiome Research, School of Biomedical Sciences, Translational Research Institute, Queensland University of Technology (QUT), Woolloongabba, Queensland, Australia; Australian Centre for Water and Environmental Biotechnology, Faculty of Engineering, Architecture and Information Technology, The University of Queensland, St Lucia, Queensland 4072, Australia

**Keywords:** nitrate, ethane, butane, *Ca.* A. nitratireducens

## Abstract

The short-chain gaseous alkanes (ethane, propane, and butane; SCGAs) are important components of natural gas, yet their fate in environmental systems is poorly understood. Microbially mediated anaerobic oxidation of SCGAs coupled to nitrate reduction has been demonstrated for propane, but is yet to be shown for ethane or butane—despite being energetically feasible. Here we report two independent bacterial enrichments performing anaerobic ethane and butane oxidation, respectively, coupled to nitrate reduction to dinitrogen gas and ammonium. Isotopic ^13^C- and ^15^N-labelling experiments, mass and electron balance tests, and metabolite and meta-omics analyses collectively reveal that the recently described propane-oxidizing “*Candidatus Alkanivorans nitratireducens*” was also responsible for nitrate-dependent anaerobic oxidation of the SCGAs in both these enrichments. The complete genome of this species encodes alkylsuccinate synthase genes for the activation of ethane/butane via fumarate addition. Further substrate range tests confirm that “*Ca. A. nitratireducens*” is metabolically versatile, being able to degrade ethane, propane, and butane under anoxic conditions. Moreover, our study proves nitrate as an additional electron sink for ethane and butane in anaerobic environments, and for the first time demonstrates the use of the fumarate addition pathway in anaerobic ethane oxidation. These findings contribute to our understanding of microbial metabolism of SCGAs in anaerobic environments.

## Introduction

Short-chain gaseous alkanes (SCGAs), including ethane, propane, and butane, are abundant components of natural gas (up to 20%) and contribute significantly to the formation of tropospheric ozone and secondary organic aerosols [1-3], thus negatively impacting air quality and climate [4, 5]. The atmospheric SCGA emissions have greatly increased since preindustrial times, reaching ~10 Tg year^−1^ for ethane, propane, butane and ~4 Tg year^−1^ for *iso*-butane [6, 7]. Microorganisms can utilize the SCGAs under oxic and anoxic conditions, significantly reducing their flux from natural ecosystems to the atmosphere [8, 9].

Although the microbiology of aerobic oxidation of SCGAs has been well studied [10], the microorganisms and metabolic pathways involved in the anaerobic oxidation of these gases have only been identified in recent years. The archaeal species “*Candidatus Argoarchaeum ethanivorans*” and “*Candidatus Syntrophoarchaeum*” oxidize ethane and butane via the formation of ethyl- or butyl-coenzyme M, respectively, in syntrophic consortia with sulphate-reducing bacteria (SRB) [11, 12]. In contrast, the deltaproteobacterial isolate *Desulfosarcina aeriophaga* BuS5 oxidizes propane and butane via a reaction with fumarate, generating propyl- and butyl-succinates (the fumarate addition pathway), coupled with the direct reduction of sulphate to sulphide [13, 14]. The bacterium “*Candidatus Methylomirabilis oxyfera*” was also shown to be able to degrade ethane and propane, although it remains unknown whether these carbon sources support continuous growth [15]. Moreover, our recent study described a bacterial species “*Candidatus Alkanivorans nitratireducens*” belonging to the Class of *Symbiobacteriia* that can oxidize propane via the fumarate addition pathway coupled to the reduction of nitrate to nitrite [16]. The oxidation of ethane and butane coupled to nitrate reduction is yet to be shown, but would also be thermodynamically feasible (Equations (1) and (2)) and potentially important given the prevalence of nitrate in natural environments [17, 18].


(1) \begin{equation*} \kern-.2pc{\mathrm{C}}_2{\mathrm{H}}_6+{{7\mathrm{NO}}_3}^{-}\to{2\mathrm{CO}}_2+{{7\mathrm{NO}}_2}^{-}+{3\mathrm{H}}_2\mathrm{O}\kern1.5pc \Delta{\mathrm{G}}^{{\mathrm{o}}^{{\prime}}}=-949\ \mathrm{kJ}/\mathrm{mol}\ {\mathrm{C}}_2{\mathrm{H}}_6 \end{equation*}

(2) \begin{equation*} {\mathrm{C}}_4{\mathrm{H}}_{10}+13{{\mathrm{NO}}_3}^{-}\to{4\mathrm{CO}}_2+13{{\mathrm{NO}}_2}^{-}+{5\mathrm{H}}_2\mathrm{O}\kern1.25em {\Delta \mathrm{G}}^{{\mathrm{o}}^{{\prime}}}=-1752\ \mathrm{kJ}/\mathrm{mol}\ {\mathrm{C}}_4{\mathrm{H}}_{10} \end{equation*}

Anaerobic ethane oxidation remains poorly understood, with direct evidence for this metabolic process limited to archaea [11, 19]. Indeed, ethane activation mediated by bacteria has not been proven, in clear contrast to the multiple discoveries of SRB-mediated anaerobic propane and butane degradation [13, 20, 21]. The fumarate addition pathway is considered the most common mechanism for anaerobic degradation of hydrocarbons including propane, butane, and various other *n*-alkanes ranging from C_6_ (*n*-hexane) to C_16_ (*n*-hexadecane) [22-25]. The oxidation of ethane via this mechanism is also likely to occur in the environment, given ethyl-succinate, the signature metabolite generated by ethane activation via reaction with fumarate, is frequently detected in hydrocarbon-rich environments, such as crude oil production wells, coal beds, and oilfields [26-28]. However, physiological evidence for anaerobic ethane oxidation via the fumarate addition pathway is lacking.

In this study, we address knowledge gaps by enriching microbial consortia able to couple anaerobic ethane and butane oxidation to nitrate reduction, and characterizing the key metabolic pathways via a multi-omics approach (metagenomics, metatranscriptomics, and metaproteomics). The alkane-oxidizing population in both enrichments is the same species as the anaerobic propane-degrading bacteria “*Ca. A. nitratireducens*” identified previously [16], and is suggested to mediate ethane and butane oxidation via reactions with fumarate.

## Results and discussion

### Enrichment cultures able to mediate nitrate-dependent anaerobic oxidation of ethane and butane

Two anaerobic bioreactors seeded with activated sludge and anaerobic digestion sludge from a wastewater treatment plant were operated for more than 1000 days. One was fed with ethane (C_2_H_6_) and nitrate, while the other with butane (C_4_H_10_) and nitrate. The C_2_H_6_-fed bioreactor showed simultaneous consumption of C_2_H_6_ and nitrate, resulting in the production of dinitrogen gas and ammonium, along with transient accumulation of nitrite (Supplementary Fig. 1A). Similarly, nitrate consumption and ammonium production were observed in the C_4_H_10_-fed reactor (Supplementary Fig. 1B). No nitrate consumption was observed in the control incubations without the addition of either C_2_H_6_ or C_4_H_10_ or enrichment culture biomass (Supplementary Fig. 2), indicating that nitrate reduction (to nitrite, dinitrogen gas, and ammonium) was a biological process and coupled to the consumption of these alkanes.

**Figure 1:**
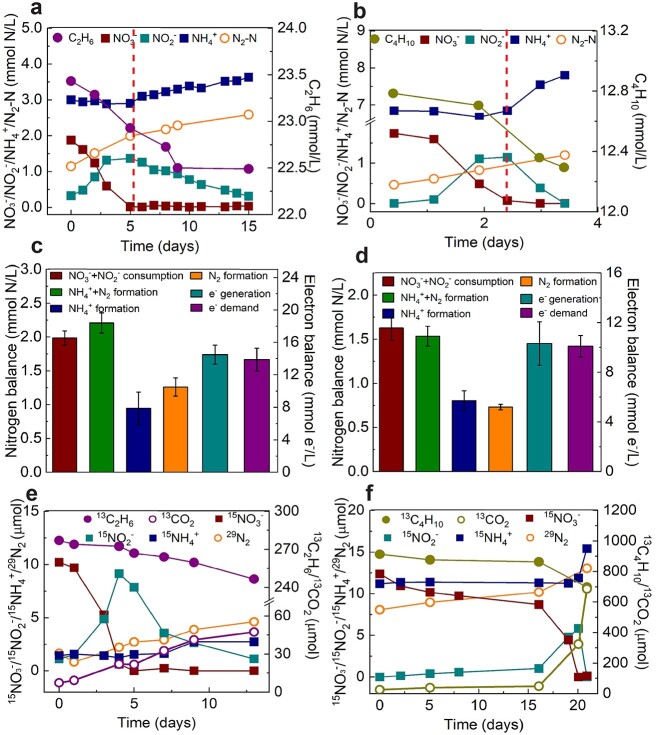
Mass and electron balance batch tests, along with isotope labelling experiments, confirmed that anaerobic ethane/butane oxidation was coupled to nitrate reduction by the bioreactor enrichment cultures fed with C_2_H_6_/C_4_H_10_; (A, B) typical biochemical profiles of the ethane (A, started on Day 490) and butane (B, started on Day 1100) systems showing simultaneous nitrate and ethane/butane consumption with transitory formation of nitrite, and production of dinitrogen gas and ammonium; there were two distinct phases for NO_3_^−^ reduction; in Phase 1, NO_3_^−^ was reduced to NO_2_^−^ and N_2_, with negligible NH_4_^+^ production, while in Phase 2, the accumulated NO_2_^−^ was reduced to both N_2_ and NH_4_^+^; (C, D) average nitrogen- and electron balances calculated from the three batch tests for C_2_H_6_- (C) and C_4_H_10_- (D) fed bioreactors (Supplementary Table 1 shows the complete data and calculation); error bars represent standard errors from biological triplicates; oxidation of ^13^C_2_H_6_ (E) or ^13^C_4_H_10_ (F) to ^13^CO_2_, and reduction of ^15^NO_3_^−^ to ^15^NH_4_^+^ and ^29^N_2_ with temporary generation of ^15^NO_2_^−^ during the isotope labelling test.

Stoichiometric experiments were conducted directly in the parent C_2_H_6_-fed reactor or with subcultures from the parent C_4_H_10_-fed reactor to establish nitrogen and electron balances. The reduction of NO_3_^−^ proceeded in two distinct phases for both C_2_H_6_- and C_4_H_10_-fed systems (Fig. 1A and B, Supplementary Fig. 3). In Phase 1, NO_3_^−^ was reduced to NO_2_^−^ and N_2_ with negligible NH_4_^+^ accumulation (Equations (1), (2), (4), and (5)). In Phase 2, when NO_3_^−^ was depleted, NO_2_^−^ was further reduced to NH_4_^+^ and N_2_ (Equations (2), (3), (5), and (6)). The total amounts of the produced nitrogen species (NH_4_^+^ + N_2_) for C_2_H_6_- (2.21 ± 0.15 mmol N/l) and C_4_H_10_-fed (1.53 ± 0.08 mmol N/l) batch tests were close to the amounts of nitrogen oxyanions consumed (NO_3_^−^ + NO_2_^−^, 1.98 ± 0.10, and 1.63 ± 0.10 mmol N/l for C_2_H_6_ and C_4_H_10_-fed cultures, respectively, Fig. 1C and D, Supplementary Table 1). This indicates that NH_4_^+^ and N_2_ were the final products generated from NO_3_^−^ and NO_2_^−^ reduction. The amounts of electrons required for denitrification (NO_3_^−^ reduction to N_2_) and dissimilatory nitrate reduction to ammonia (DNRA) in the C_2_H_6_- and C_4_H_10_-fed batch tests represent 96 ± 2% and 99 ± 6% of the maximum electrons available in C_2_H_6_ and C_4_H_10_ oxidation to CO_2_, respectively (Fig. 1C and D, Supplementary Table 1), suggesting electrons were mainly diverted to NO_3_^−^ reduction in these systems.

To verify the final products of anaerobic C_2_H_6_/C_4_H_10_ oxidation coupled to nitrate reduction, subcultures from the parent reactors were incubated with ^13^C-labelled C_2_H_6_ (^13^CH_3_^13^CH_3_) or C_4_H_10_ (^13^CH_3_^13^CH_2_^13^CH_2_^13^CH_3_) and ^15^N-labelled nitrate (^15^NO_3_^−^) in 0.6 l glass vessels. Concomitant to ^13^C_2_H_6_/^13^C_4_H_10_ consumption, ^13^CO_2_ was produced in both tests. The amounts of ^13^CO_2_ produced from the labelled C_2_H_6_- (40 μmol) and C_4_H_10_-fed (661 μmol) batches were 67% and 77%, respectively, of the consumed ^13^C in ^13^C_2_H_6_ (60 μmol) and ^13^C_4_H_10_ (840 μmol) (Fig. 1E and F). Similarly, the total amounts of CO_2_ produced were 71% and 83% of total consumed carbon in C_2_H_6_ and C_4_H_10_, respectively (Supplementary Fig. 4). These results suggest that CO_2_ was the dominant end product from C_2_H_6_ and C_4_H_10_ oxidation, while a minor fraction of carbon from SCGAs was likely assimilated into biomass. The total ^15^N in ^29^N_2_, ^30^N_2_, and ^15^NH_4_^+^ produced (8.9 and 9.2 μmol in total in the C_2_H_6_ and C_4_H_10_-fed batch, respectively) was concordant with the totally consumed ^15^NO_3_^−^ (10.2 and 12.2 μmol for C_2_H_6_ and C_4_H_10_-fed batches, respectively), confirming the reduction of NO_3_^−^ to N_2_ and NH_4_^+^ (Fig. 1E and F). These findings collectively support nitrate-dependent anaerobic oxidation of C_2_H_6_ and C_4_H_10_ in the two bioreactors (Equations (1)–(6)).


(3) \begin{equation*}\footnotesize{3\mathrm{C}}_2{\mathrm{H}}_6+14{{\mathrm{NO}}_2}^{-}+14{\mathrm{H}}^{+}\to{6\mathrm{CO}}_2+{7\mathrm{N}}_2+16{\mathrm{H}}_2\mathrm{O}\ \ \ \ \ {\Delta \mathrm{G}}^{{\mathrm{o}}^{{\prime}}}=-1661\ \mathrm{kJ}/\mathrm{mol}\ {\mathrm{C}}_2{\mathrm{H}}_6\\ \end{equation*}

(4) \begin{equation*} \footnotesize{3\mathrm{C}}_2{\mathrm{H}}_6+{{7\mathrm{NO}}_2}^{-}+14{\mathrm{H}}^{+}\to{6\mathrm{CO}}_2+{{7\mathrm{NH}}_4}^{+}+{2\mathrm{H}}_2\mathrm{O}\ \ \ \\ \Delta{\mathrm{G}}^{{\mathrm{o}}^{{\prime}}}=-826\ \mathrm{kJ}/\mathrm{mol}\ {\mathrm{C}}_2{\mathrm{H}}_6\ \ \ \ \end{equation*}

(5) \begin{equation*} \footnotesize{3\mathrm{C}}_4{\mathrm{H}}_{10}+26{{\mathrm{N}\mathrm{O}}_2}^{-}+26{\mathrm{H}}^{+}\to 12{\mathrm{C}\mathrm{O}}_2+13{\mathrm{N}}_2+28{\mathrm{H}}_2\mathrm{O}\ \ \Delta{\mathrm{G}}^{{\mathrm{o}}^{{\prime}}}=-3105\ \mathrm{kJ}/\mathrm{mol}\ {\mathrm{C}}_4{\mathrm{H}}_{10} \end{equation*}

(6) \begin{equation*}\footnotesize{3\mathrm{C}}_4{\mathrm{H}}_{10}+13{{\mathrm{NO}}_2}^{-}+26{\mathrm{H}}^{+}\to 12{\mathrm{C}\mathrm{O}}_2+13{{\mathrm{NH}}_4}^{+}+{2\mathrm{H}}_2\mathrm{O}\\, \Delta{\mathrm{G}}^{{\mathrm{o}}^{{\prime}}}=-1555\ \mathrm{kJ}/\mathrm{mol}\ {\mathrm{C}}_4{\mathrm{H}}_{10} \end{equation*}

### Microbial community structure and genome recovery

16S rRNA gene amplicon sequencing of the biomass from both bioreactor enrichments revealed the dominance of the recently described propane oxidizing firmicute “*Ca. A. nitratireducens*” [16] in both systems (100% amplicon sequence similarity; 5.3%–10.5% abundance for C_2_H_6_-fed reactor and 4.3%–18.3% for C_4_H_10_-fed reactor, Supplementary Fig. 5). The metagenomes of both cultures were obtained by applying both long (Nanopore) and short read (Illumina) sequencing for biomass samples collected from the C_2_H_6_- (on Day 746) and C_4_H_10_-fed (Day 1150) bioreactors (Supplementary Table 2). In total, 63 and 37 high-quality genomes (≥70% completeness and ≤ 10% contamination based on CheckM) were retrieved for the C_2_H_6_- and C_4_H_10_-fed bioreactor enrichments, respectively (Supplementary Data 1). These included two complete circularized genomes of the dominant “*Ca*. *A. nitratireducens*” in the C_2_H_6_- (15.0% of relative abundance, a size of 2.42 Mbp, Supplementary Table 3, Supplementary Fig. 6) and C_4_H_10_-fed (16.7% of relative abundance, a size of 2.32 Mbp, Supplementary Table 4, Supplementary Fig. 6) bioreactors. These metagenome-assembled genomes (MAGs) had average nucleotide identities of 99.96% and 99.55%, and average amino acid identities (AAI) of 99.96% and 99.57% (Supplementary Fig. 7) to the “*Ca. A. nitratireducens*” genome previously recovered from the C_3_H_8_-fed culture [16], confirming that the three genomes likely represent the same species [29]. Other dominant species include *Patescibacteria* and *Fimbriimonadaceae* in the C_2_H_6_-fed reactor, and *Promineofilaceae*, *Phycisphaerales*, and *Anaerolineales* in the C_4_H_10_-fed reactor (Supplementary Tables 3 and 4). Further annotation of MAGs for these bacteria suggest that they do not contain genes known to facilitate anaerobic SCGA oxidation, including genes for alkylsuccinate synthase (AssA) and alkyl-coenzyme M reductase, indicating that they unlikely play a direct role in SCGA metabolism. The metabolic activity and potential of these bacteria require further investigation.

### Analyses of metabolic pathways of “*Ca*. *A. nitratireducens*”

**Figure 2:**
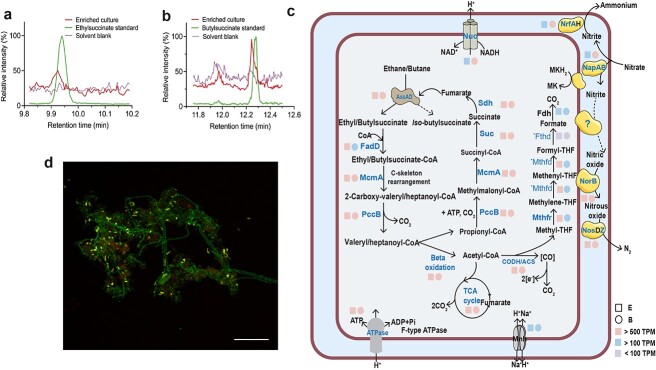
Metabolic intermediates, inferred metabolic pathways, and a fluorescent micrograph of “*Ca. A. nitratireducens*”; (A) partial ion chromatograms (ion transition, *m*/*z*: 275>73.1) of culture extracts from the C_2_H_6_-fed bioreactor displayed a characteristic peak at a retention time of 9.940 min, matching the ethylsuccinate standard; (B**)** a characteristic peak at a retention time of 12.245 min (ion transition, *m*/*z*: 303.0>147.1), consistent with the peak from the butyl-succinate standard, was observed for the culture extracts from the C_4_H_10_-fed bioreactor (*n* = 4 at different sampling points); (C) cell cartoon illustrating “*Ca. A. nitratireducens*” in the C_2_H_6_- or C_4_H_10_-fed bioreactors (E or B) use AssA to activate ethane/butane to ethyl/butyl-succinate, which are further converted to acetyl-CoA and propionyl-CoA; fumarate could be regenerated by the methylmalonyl-CoA pathway or the TCA cycle; CO_2_ is produced through the TCA cycle or the reverse WL pathway; the E and B both harbour genes that enable denitrification (except *nirS/K*) and dissimilatory nitrate reduction to ammonium; the colour of the square and circle symbols indicates the normalized gene expression values calculated as TPM (total TPM); blue bold text shows that the proteins were fully or partially detected in the protein extracts (^*^Mthfd and ^*^Fthd were only identified in B and E, respectively), while proteins in black text were not detected; **(**D) a composite fluorescence micrograph of the C_4_H_10_-fed enrichment culture hybridized with the SYMB-1018 probe [16] (Cy3, red; targeting “*Ca. A. nitratireducens*”) and EUBmix probe set [37] (fluorescein isothiocyanate label, green; all bacteria); “*Ca. A. nitratireducens*” cells appear yellow (red + green) and other bacterial cells appear green; the scale bar indicates 20 μm; the representative image was selected based on the visual assessment of >3 separate hybridization experiments; FISH was performed as detailed in our previous study [16].

Consistent with “*Ca. A. nitratireducens*” originating from the C_3_H_8_-fed system (referred to as MAG/population P), the closed genomes of “*Ca. A. nitratireducens*” in C_2_H_6_- and C_4_H_10_-fed bioreactors (referred to as MAGs/population E and B) both contain three alkylsuccinate synthase catalytic subunits (Supplementary Fig. 8A), which are phylogenetically distant from other available fumarate addition enzymes in the UniProt database (Supplementary Fig. 8B). A search of the metagenome libraries confirmed “*Ca. A. nitratireducens*” as the only microorganism harbouring the key AssA gene. To support the role of these AssA complexes in ethane/butane oxidation, key metabolites from the active cultures were analysed by ultra-high-sensitivity triple quadrupole mass spectrometry. A mass peak (m/z: 275 > 73.1) at the retention time of 9.940 min was detected for the C_2_H_6_-fed bioreactor, corresponding to the ethyl-succinate standard (Fig. 2A). Also, a mass peak (m/z: 303.0 > 147.1) at the retention time of 12.245 min was detected for the C_4_H_10_-fed bioreactor, corresponding to the butyl-succinate standard (Fig. 2B). These findings support that ethane/butane were activated by addition of fumarate, thus generating ethyl/butyl-succinate, which is consistent with the action of AssA.

MAGs E and B also harbour other key genes involved in the further degradation of ethyl/butyl-succinate, including the methylmalonyl-CoA mutase genes (*mcmA*) for carbon-skeleton rearrangement, the propionyl-CoA carboxylase genes (*pccB*) for decarboxylation, and the genes for beta-oxidation (Supplementary Data 2 and 3, Fig. 2C). The propionyl-CoA generated from beta-oxidation could enter the methylmalonyl-CoA pathway to regenerate fumarate for subsequent rounds of ethane/butane activation. The acetyl-CoA may be completely oxidized to CO_2_ or used for fumarate regeneration via the oxidative tricarboxylic acid (TCA) cycle. CO_2_ can also be generated by the oxidation of acetyl-CoA through the reverse Wood–Ljungdahl (WL) pathway for MAGs E and B (Supplementary Data 2 and 3, Fig. 2C), consistent with that proposed for MAG P and the sulphate-dependent propane oxidizer—*D. aeriophaga* BuS5 [14, 16]. The metatranscriptomic and metaproteomic data indicated that MAGs E and B had associated expression of the proposed fumarate addition pathway for complete ethane/butane oxidation to CO_2_ after alkane additions (Supplementary Data 2 and 3, Fig. 2C). The “*Ca. A. nitratireducens*” dominated the transcriptome profile of both the C_2_H_6_- (61.5% of the total transcriptome reads, Supplementary Table 5) and C_4_H_10_-fed bioreactors (84.5% the total transcriptome reads, Supplementary Table 6), while the relative activities of other co-existing microbial population were substantially lower. This indicates that “*Ca. A. nitratireducens*” are the key drivers of anaerobic alkane oxidation in these systems.

Similar to MAG P, the MAGs E and B both encode genes encoding nitrate reductase (*napAB*) and cytochrome *c* nitrite reductases (*nrfAH*) required for DNRA process, which are all expressed (Supplementary Data 2 and 3, Fig. 2C). The expression of *nrfAH* was much higher for populations B than E in Phase 2, consistent with the significantly higher DNRA rates (*P* < 0.05) in the C_4_H_10_-fed bioreactor (0.77 ± 0.27 mmol/l/day) compared to the C_2_H_6_-fed bioreactor (0.11 ± 0.08 mmol/l/day). The NapAB and NrfA were also identified in protein extracts from both the C_2_H_6_- and C_4_H_10_-fed cultures (Supplementary Data 2 and 3, Fig. 2C), further supporting that populations E and B were performing DNRA in these systems. Other members of the communities also expressed genes for DNRA but at much lower levels compared to E and B MAGs (Supplementary Data 4). The closed E and B MAGs both lack nitric oxide-producing nitrite reductase (*nirS/K*) but encode nitric oxide reductase (*norB*) and nitrous oxide reductase (*nosZD*), which is consistent with MAG P. The *norB* and *nosD* genes were expressed and detected in the protein extracts for both the E and B populations (Supplementary Data 2 and 3, Fig. 2C), suggesting the active roles of these populations in the reduction of nitric oxide to dinitrogen gas. The phenomena that dinitrogen gas was generated without the apparent involvement of *nirS/K* for the dominant “*Ca. A. nitratireducens*” in all three systems indicates that this species may indeed utilize a novel gene or novel pathway to reduce nitrite to nitric oxide [16]. However, we cannot completely rule out the possibity that other microorganims like *Fimbriimonadaceae*, *Burkholderiales*, and *Promineofilum* in the ethane reactor, and *Phycisphaerales*, *Anaerolineales*, and *Promineofilaceae* in the butane reactor, may also contribute to nitrite reduction to nitric oxide or denitrification to dinitrogen gas, given that they express *nirS/K* and other denitrification genes (Supplementary Data 4). To further confirm the exact metabolic pathways of “*Ca. A. nitratireducens*” for nitrogen and carbon transformations, pure culture isolation is likely needed.

### Short-chain gaseous alkane metabolic versatility of “*Ca*. *A. nitratireducens*”

Structural modelling and molecular dynamics (MD) simulations were conducted to understand the potential functions of different AssAs in “*Ca. A. nitratireducens.*” MAG E encodes three AssAs that are 852aa in length with differing AAI between them (90.96% and 96.60%, Supplementary Table 7). The shorter AssA genes identified in the P and B MAGs for “*Ca. A. nitratireducens*” were found to be due to open reading frame calling issues [30] via full length alignments of E MAG AssA genes to the AssA regions of the P and B MAGs, along with manual identification of the start and stop codon. Further analyses of the AssAs in three MAGs show that full length alignment to the conserved domain (cd01677) for pyruvate formate lyase 2 and related enzymes is only found in the 852aa AssAs [31], suggesting these AssAs are more likely to be complete. Given the overall high AssA gene similarities between MAGs (Supplementary Table 8), the three complete AssA genes in MAG E were used for structural modelling and MD simulations.

**Figure 3:**
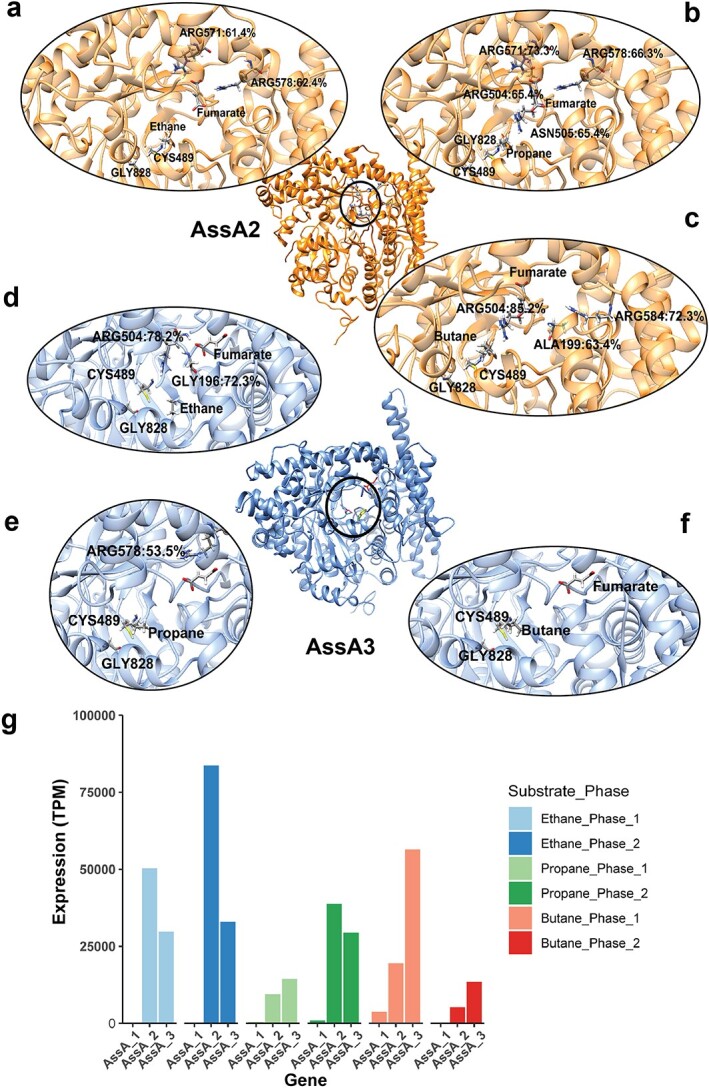
The MD simulations and gene expression of AssAs in “*Ca. A. nitratireducens*”; the structural representations of binding complexes of AssA2 with fumarate and ethane (A)/propane (B)/butane (C), and AssA3 with fumarate and ethane (D)/propane (E)/butane (F); key residues of Cys489 and Gly828 are close to each other in all systems; residues with occupancy of hydrogen bonds >50% were also included in the figures; (g) the normalized gene expression values of assA genes in “*Ca. A. nitratireducens*” from C_2_H_6_-, C_3_H_8_-, and C_4_H_10_-fed systems (calculated as total TPM).

The MD results suggest that AssA1 cannot stably bind to the key substrate—fumarate (Supplementary Movie S1), while AssA2 and AssA3 can form stable binding complexes with fumarate and ethane/propane/butane (Supplementary Movie S2–S7, Fig. 3A–F, Supplementary Fig. 9). Hydrogen bonding networks were found to be critical for the SCGA and fumarate bindings (Fig. 3A–F, Supplementary Fig. 10). In addition, the putative radical sites Cys489 and Gly828 are situated at the core of AssA2/AssA3 and close to each other in all binding complexes (Fig. 3A–F). These characteristics were suggested to be important for radical transfers in glycyl radical enzymes [32, 33], indicating that the radical transfer pathway may govern fumarate addition in AssA2/AssA3.

Metatranscriptomic profiles of the ethane, propane, and butane systems were mapped onto E MAG to ensure consistency of the AssA gene lengths. In support of the MD results, AssA1 are relatively lowly expressed in all systems (Fig. 3G). However, the expression levels of AssA2 and AssA3 are relatively high in all systems (Fig. 3G), suggesting these proteins are more likely responsible for SCGA activation by “*Ca. A. nitratireducens.*”

**Figure 4:**
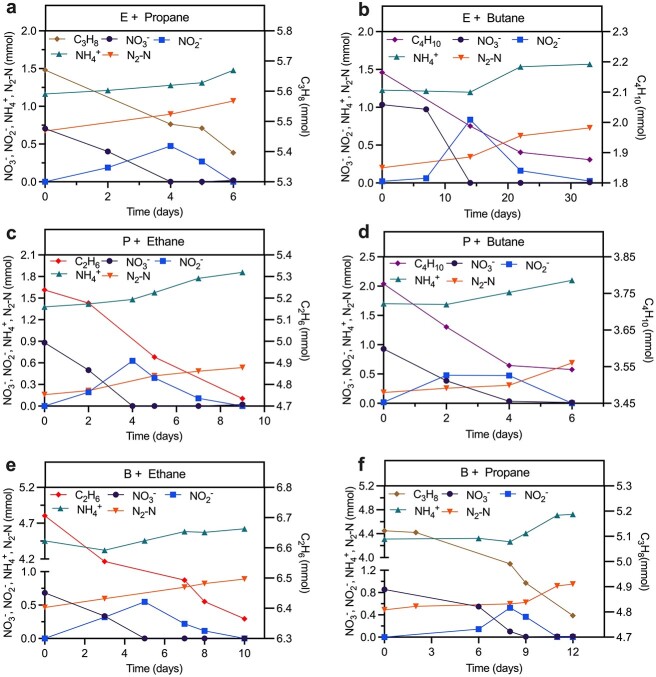
Substrate range tests for the “*Ca. A. nitratireducens*” enriched in the C_2_H_6_-, C_3_H_8_-, and C_4_H_10_-fed bioreactors; subculture from the ethane bioreactor supplemented with propane (A) or butane (B) showed simultaneous nitrate and propane/butane consumption with production of dinitrogen gas and ammonium; the same was observed for subcultures from the propane bioreactor supplemented with ethane (C) and butane (D), and the butane bioreactor provided with ethane (E) and propane (F); each test was conducted in triplicate (results of other tests were included in Supplementary Fig. 11).

To further validate if “*Ca. A. nitratireducens*” is indeed able to oxidize all three SCGAs, substrate range tests were conducted for the C_2_H_6_-, C_3_H_8_-, and C_4_H_10_-fed cultures. Incubation of subcultures from the C_2_H_6_-, C_3_H_8_-, and C_4_H_10_-fed bioreactors with the other two SCGAs showed obvious ethane/propane/butane oxidation coupled to nitrate reduction to dinitrogen gas and ammonium (Fig. 4A–F, Supplementary Fig. 11). These results provide evidence that “*Ca. A. nitratireducens*” has the metabolic versatility to oxidize the three tested SCGAs using nitrate as a terminal electron acceptor.

### Implications

This study identified “*Ca*. *A. nitratireducens*” as a metabolically diverse anaerobic SCGA oxidizer able to utilize ethane, propane, and butane. In previous studies, SRB affiliated with the *Desulfosarcina–Desulfococcus* cluster and the archaeon *Candidatus* “*Syntrophoarchaeum*” were suggested to be only capable of oxidizing propane and butane but not ethane [12, 13, 20]. Conversely, the archaeon *Candidatus* “*Ethanoperedens thermophilum*” could only oxidize ethane [19]. Importantly, this study identified a bacterium performing anaerobic ethane oxidation, previously known for archaea only. This study also provides the first physiological evidence for the involvement of the fumarate addition pathway in anaerobic ethane oxidation, closing a key knowledge gap in our understanding of anaerobic SCGA oxidation.

Furthermore, the newly discovered nitrate-dependent anaerobic ethane and butane oxidation (n-DAEO/n*-*DABO) indicate that nitrate is an additional electron sink for C_2_H_6_ and C_4_H_10_, potentially contributing to reducing the negative impacts of C_2_H_6_ and C_4_H_10_ on air quality and on climate. C_2_H_6_ and C_4_H_10_ are recognized as indirect greenhouse gases with net global warming potentials of 10 and 7 times, respectively, that of CO_2_ (100-year horizon) [34]. Moreover, they also contribute to the production of hazardous substances including carbon monoxide and peroxyacetyl nitrate [35], which are significant air pollutants. This research advances our understanding of the role of microorganisms in constraining SCGA emissions by identifying another microbially mediated link between the global carbon and nitrogen cycles. Considering the widespread presence of nitrate and rising emissions of nonmethane SCGAs caused by oil and natural gas exploitation [9], “*Ca*. *A. nitratireducens*” may play an important role in global carbon and nitrogen cycling.

## Methods

### Bioreactor setup and operation

Activated sludge (50 ml) and anaerobic digestion sludge (100 ml) from a full-scale wastewater treatment plant (Luggage Point, Brisbane, Australia) were used as inoculum for the ethane and *n*-butane (hereafter butane) bioreactor enrichment. This choice of inoculum was based on previous successful enrichment of anaerobic propane degradation bacteria from this source and the small quantities of ethane and butane detected in anaerobic digestion systems [36]. The incubations with ethane or butane as a sole carbon source were set up in a lab bioreactor with a volume of 1.12 and 2.3 l, respectively. An anoxic mineral medium [16] of 0.67 and 1.69 l was initially added to the ethane and butane reactor (~1:4.5 and 1:11.3 of sludge to medium ratios, respectively), leaving a headspace of 0.3 and 0.46 l, respectively. The ethane/butane reactors were periodically flushed with pure ethane/butane gas (99.99%, Coregas, Australia) to maintain the ethane/butane partial pressure in the headspace between 0.9 and 1.2 atm. A concentrated stock solution (80 g NO_3_^−^N l^−1^) was manually pulse-fed to the reactors to replenish NO_3_^−^ to 20–30 mg N l^−1^. The bioreactors were continuously mixed using a magnetic stirrer (IKA, Labtek, Australia) at 650 rpm and operated in a thermostatic chamber (35 ± 1°C). Every 1–4 months, the stirrers were stopped for 24 h to allow biomass to settle, and the supernatant of 0.2–0.8 l was then replaced with fresh medium. The pH was manually adjusted to 6.8–7.5 using a 1 M anoxic HCl solution. Liquid samples (0.4–0.6 ml each) were collected periodically (2–5 samples per week) and filtered immediately using a 0.22 μm membrane filter (polyethersulfone filter, Millex, USA) for the analysis of NO_3_^−^, NO_2_^−^, and NH_4_^+^. A gas sample (100 μl) from the headspace was withdrawn regularly (three to five times per week) using a gas-tight syringe (1710 SLSYR, Hamilton) for the determination of C_2_H_6_ and N_2_.

### Batch tests for nitrogen and electron balances

Stoichiometric tests were carried out *in situ* for the biomass of the 1.12 l ethane parent reactor on Days 490, 522, and 559 to investigate nitrogen and electron balances. For stoichiometry determination of nitrate reduction coupled to anaerobic butane oxidation, triplicate batch tests were conducted in 650 ml glass vessels with a subsample of 500 ml biomass anaerobically transferred from the 2.3 l butane parent bioreactor. Total amounts of ethane/butane and N_2_ were calculated by considering ethane/butane/N_2_ in both the headspace (monitored) and liquid phase (calculated with Henry’s law). Two negative control groups were set up in 600 ml bottles: (i) control groups containing only enriched cultures and nitrate (ethane/butane was removed by flushing the bottles with pure argon gas for 20 min); (ii) abiotic control groups without enriched cultures (only synthetic medium containing ethane/butane and nitrate was provided).

### Isotope labelling experiment

A 480 ml subculture from the ethane/butane bioreactor was transferred to a 600 ml glass vessel. The ethane culture was flushed with pure C_2_H_6_ for 10 min, and the 5 ml ^13^C-labelled C_2_H_6_ (^13^CH_3_^13^CH_2_,99 atom % ^13^C, Sigma) was injected into the headspace, followed by an introduction of 0.12 ml nitrate stock solution (40 g N l^−1^), which contained ~1% ^15^N-labelled NO_3_^−^ (98 atom % ^15^N, Sigma). The butane culture was flushed with argon gas (99.99%, Coregas, Australia) for 20 min. Approximately 24 ml ^13^C-labelled butane (^13^CH_3_^13^CH_2_^13^CH_2_^13^CH_3_, 99 atom% ^13^C, Sigma) was injected into the headspace through the septum. Approximately 1 ml nitrate stock solution (10 g N l^−1^) containing ~1% ^15^N-labelled sodium nitrate (98 atom % ^15^N, Sigma) was added to achieve a concentration of ~20 mg N l^−1^. Liquid samples were collected (two to five samples per week) and filtered through 0.22 μm filters for analyzing soluble nitrogen species and respective isotopic fractions. Gaseous samples were collected (four to seven samples in total) from the headspace using a gas-tight syringe (model 1710 SL SYR, Hamilton, USA) and injected into helium-flushed exetainer vials (Labco, UK) for measuring total C_2_H_6_, C_4_H_10_, CO_2_, and N_2_ in gas phases and their isotopic fractions. For the measurement of the dissolved CO_2_, ~0.5 ml liquid samples were collected and injected into vacuum vials, followed by acidification with HCl stock solution (1 M), and settled for at least 0.5 h to achieve gas–liquid equilibrium before CO_2_ quantification.

### Substrate range tests for “*Ca*. *A. nitratireducens*”

To examine whether the C_2_H_6_-fed culture has the capability of oxidizing propane and butane, two batch tests were setup by mixing 200 ml culture from the C_2_H_6_-fed bioreactor with 280 ml anoxic mineral medium in 600 ml glass vessels. The two batch reactors were then flushed with pure propane and butane gases, respectively, to remove dissolved ethane and provide propane and butane. The nitrate stock solution (10 g N l^−1^) was added to the reactors to achieve an initial concentration of ~20 mg N l^−^. The batch tests were conducted in triplicate. Liquid and gas samples were collected as described above. Similarly, cultures from the parent C_3_H_8_- or C_4_H_10_-fed bioreactor were also transferred to new batch reactors and then incubated with ethane and butane, or ethane and propane.

### Chemical analysis

Soluble nitrogen species (NO_3_^−^, NO_2_^−^, and NH_4_^+^) and gas components including C_2_H_6_, CO_2_, and N_2_ in the headspace were determined as described previously [16]. The butane, ^13^C-labelled butane, ^13^C-labelled ethane, ^13^CO_2_, ^29^N_2_, and ^30^N_2_ in gaseous samples were quantified using a GC (7890A, Agilent, USA) coupled to a quadrupole mass spectrometer (MS, 5957C inert MSD, Agilent, USA). The GC–MS was operated as described in the supplementary text.

The isotopic fractions of ^15^N-labelled nitrogen-oxyanions (NO_3_^−^ + NO_2_^−^) were analysed using a Thermo Delta V isotope ratio mass spectrometer (IRMS; Thermo Fisher Scientific, USA) following conversion to N_2_O via the denitrifier protocol [38]. To measure ^15^N-labelled NO_3_^−^, NO_2_^−^ was removed from the liquid samples with 4% (wt/vol) sulfamic acid in 10% HCl as described previously [39]. The fraction of ^15^N in NO_2_^−^ was calculated according to the difference between ^15^N fraction in nitrogen-oxyanions (NO_3_^−^ + NO_2_^−^) and that in NO_3_^−^. To analyse ^15^N-labelled NH_4_^+^, NH_4_^+^ was trapped in GF/D filters (Whatman, UK) with a microdiffusion method [40] and then combusted before IRMS analysis.

### Metagenomic sequencing, and recovery and assessment of microbial populations

Biomass collected on Day 746 and 1150 for ethane and butane bioreactors, respectively, were used for short- and long-read metagenomic sequencing as described in the supplementary text. Pair-end short reads were trimmed using ReadTrim (https://github.com/jlli6t/ReadTrim) with parameter –-remove_dups–-minlen 100″. Nanopore sequencing signals were processed using MinKNOW 20.06.18 and base-called using Guppy 4.0.11 (https://community.nanoporetech.com/), resulting in 53.8 million reads with quality >Q7 with N50 of 2.55 kb. Adapters were trimmed using Porechop v0.2.4 (https://github.com/rrwick/Porechop).

Assembly and binning were performed using Aviary (https://github.com/rhysnewell/aviary), which internally called different tools, including NanoPack [41], Flye [42], Unicycler [43], Pilon [44], Minimap2 [45], CONCOCT [46], VAMB [47], MetaBAT 1 & 2 [48, 49], MaxBin 2.0 [50], and SemiBin [51]. Specifically, hybrid assembly of short and long reads was performed using workflow “assemble.” Resulted assemblies were manually checked using Bandage [52]. Genomes of each community were then recovered using workflow “recover.” Obtained genomes were optimized and dereplicated using DASTools 1.1.2 [53]. Quality of MAGs was checked using CheckM v1.1.3 [54]. Taxonomy information of MAGs was determined using GTDB-Tk 2.1.1 [55]. Quality-trimmed short-reads were mapped to assemblies using bowtie 2.3.4.3 [56]. Coverage of genome information and other details were viewed and manually checked using IGV 2.11.1 [57]. Abundance of each MAG was profiled using CoverM 0.6.1 (https://github.com/wwood/CoverM). Genome characteristics were calculated using BioSut (https://github.com/jlli6t/BioSut).

### Functional annotation

Preliminary annotation across MAGs and unbin contigs was performed using Prokka 1.14.5 [58]. Predicted protein sequences were then searched against KEGG (July 2021) using kofamscan 1.3.0 [59], and the hit with an e-value <1e-10 and maximal F-measure was selected for each gene. UniRef100 [60] (March 2020) was searched against using diamond [61] v2.0.11.149 with “blastp—sensitive.” The best hit with e-value <1e-5 and identity >30% was selected for each gene and mapped to the KEGG Orthology database. The eggNOG v5 [62] was searched against using emapper 2.1.5 [63]. Metabolic pathways were reconstructed using KEGG. Pathways identified to be >75% complete were considered as “present.” Full-length AssA genes from the P and B MAGs were identified based on blastn hits to the AssA genes from E MAG and translated using NCBI’s Open Reading Frame (ORF) finder.

### Metatranscriptomic sequencing and data analysis

Two distinct phases were observed for the nitrate reduction in both ethane and butane bioreactors (Fig. 1A and B). For total RNA extraction, the active enriched culture (10 ml) collected from each phase was mixed with 30 ml of RNAlater solution (Sigma-Aldrich) and left to stand for 1 h before extraction. Total RNA was then extracted and sequenced as described previously [16].

The metatranscriptomic paired-end reads were mapped to dereplicated genome sets and filtered using minimum cut-off values of 97% identity and 75% alignment. The *Symbiobacteriia* MAG generated from the ethane system was selected as the representative SymBio MAG due to the presence of full length AssA genes. TranscriptM (GitHub–- sternp/transcriptm) was used to unambiguously mapp mRNA for each ORF and calculate the total transcripts per million (TPM).

### Protein extraction and metaproteomics

For protein extraction, enrichment cultures collected from Phase 1 and 2 (10 ml each phase) were pelleted by centrifugation (18 000 *g*, 4°C) and then washed with 1 × Phosphate buffered saline (PBS). The cell lysis and total protein digestion were performed as described previously [16]. The digested peptides were analysed by liquid chromatography–tandem mass spectrometry using a Dionex Ultimate 3000 RSLCnano-LC system coupled to a Q-Exactive H-X Hybrid Quadrupole-Orbitrap mass spectrometer (Thermo Scientific). Mass spectra were searched against the annotated closed MAGs of E and B, respectively, in Thermo Proteome Discoverer. The identified proteins contained at least one unique peptide with a stringency cut-off of false discovery rate (*q* value) <0.05.

### Computational analyses for catalytic subunits of different alkylsuccinate synthases in “*Ca*. *A. nitratireducens*”

#### Structural modelling and molecular dynamics simulation

The amino acid sequences of three complete AssAs were acquired from the closed genome of “*Ca*. *A. nitratireducens*” in the ethane-fed system. The tertiary structures of AssAs were modelled with Alphafold-2 [64]. Fumarate and alkanes were bound to corresponding AssA by CB-dock-2 [65]. AssA-Fumarate-Alkane complexes were solvated by CHARMM-GUI [66] with a thickness of 15 Å. Water type was TIP3P [67] and the force field was CHARMM36m [68]. NaCl (200 mM) was used to ionize the systems [69]. The final systems were then subjected to the MD simulations with NAMD 2.12 [70]. Periodic boundary condition was applied to the simulating box, and particle mesh Ewald was used for the long-range electrostatic interactions. The pressure was set at 1 atm using a Langevin thermostat with a damping coefficient of 1/ps. A Nose−́Hoover Langevin piston barostat with a decay period of 25 fs was applied. The temperature was reassigned every 500 steps. Simulations for each model include two steps. The first is 1 ns equilibration (NVP), and the second is 50 ns production run (NPT).

#### Root mean square fluctuation and deviation calculations

The root mean square fluctuation for α-carbons of the amino acid residues is calculated with Equation (1) [71, 72].


(1) \begin{equation*} {\mathrm{RMSF}}_{\mathrm{i}}={\left[\frac{1}{\mathrm{T}}\sum_{{\mathrm{t}}_{\mathrm{j}}=1}^{\mathrm{T}}|{\mathrm{r}}_{\mathrm{i}}\left({\mathrm{t}}_{\mathrm{j}}\right)-{\mathrm{r}}_{\mathrm{i}}^{\mathrm{r}\mathrm{ef}}|\right]}^{1/2} \end{equation*}

where i represents the residue ID, T represents the total simulation time (Here is the number of frames), and r_i_(t_j_) represents the position of residues i at time t_j_. The r_i_^ref^ is the reference position of residue i, calculated by the time-average position.

To measure the average distance between two protein structures, the root mean square deviation is calculated with Equation (2).


(2) \begin{equation*} \mathrm{RMSD}\left(\mathrm{t}\right)={\left[\frac{1}{\mathrm{WN}}\sum_{\mathrm{i}=1}^{\mathrm{N}}{\mathrm{w}}_{\mathrm{i}}{\left|{\mathrm{r}}_{\mathrm{i}}\left(\mathrm{t}\right)-{\mathrm{r}}_{\mathrm{i}}^{\mathrm{r}\mathrm{ef}}\right|}^2\right]}^{1/2} \end{equation*}

where W = Σw_i_ is the weighting factor, and N is the total number of atoms. The r_i_(t) is the position of atom i at time t after least square fitting the structure to the reference structure. The r_i_^ref^ is the reference position of residue i defined by the reference structure (Here we used the initial structure as the reference).

#### Hydrogen bond analyses

The hydrogen bonds were analysed by VMD [72] based on 100 frames obtained from the last MD simulations of 40–50 ns. The cut-off distance and angle for hydrogen bond analyses were set as 3.5 Å and 20°, respectively.

## Supplementary Material

Supplementary_data_1-MAG_info_wrad011

Supplementary_data_2-mapped_to_C2-C2-sym-RNA_protein_wrad011

Supplementary_data_3-mapped_to_C2-C4-sym-RNA_protein_wrad011

Supplemeentary_data_4-C2_C4-N_genes_wrad011

Movie_S1-AssA1-FMR_wrad011

Movie_S2-AssA2-FMR-C2_wrad011

Movie_S3-AssA2-FMR-C3_wrad011

Movie_S4-AssA2-FMR-C4_wrad011

Movie_S5-AssA3-FMR-C2_wrad011

Movie_S6-AssA3-FMR-C3_wrad011

Movie_S7-AssA3-FMR-C4_wrad011

Supporting_information_wrad011

## Data Availability

Sequencing data are archived in NCBI database under Project number PRJNA989758. The mass spectrometry proteomics data have been deposited to the ProteomeXchange Consortium via the PRIDE partner repository with the dataset identifier PXD039267.
