# Supplementary material for: Nitrate-driven anaerobic oxidation of ethane and butane by bacteria
Source: ISME J. 2024 Jan 10;18(1):wrad011. doi: 10.1093/ismejo/wrad011 (PMC10811727; doi:10.1093/ismejo/wrad011)
Supplement: Supporting_information_wrad011 [file supporting_information_wrad011.pdf]

Nitrate-driven anaerobic oxidation of ethane and butane by bacteria

Mengxiong Wu[1,9], Jie Li[1,9], Chun-Yu Lai[1,7,9,*], Andy O Leu[2], Shengjie Sun[3], Rui Gu[1], Dirk V Erler[4], Lian Liu[5], Lin Li[6], Gene W. Tyson[2], Zhiguo Yuan[1,8], Simon J. McIlroy[2], Jianhua Guo[1,*]

[1]Australian Centre for Water and Environmental Biotechnology, Faculty of Engineering, Architecture and Information Technology, The University of Queensland, St Lucia, Queensland 4072, Australia

[2]Centre for Microbiome Research, School of Biomedical Sciences, Queensland University of Technology (QUT), Translational Research Institute, Woolloongabba, Queensland, Australia

[3]Computational Science Program, The University of Texas at El Paso, El Paso, TX, USA

[4]Faculty of Science and Engineering, Southern Cross University, Lismore, New South Wales, Australia

[5]Metabolomics Australia (Queensland Node), Australian Institute for Bioengineering and Nanotechnology, The University of Queensland, St Lucia, QLD 4072, Australia

[6]Department of Physics, University of Texas at El Paso, El Paso, TX, USA

[7]College of Environmental and Resource Science, Zhejiang University, Hangzhou, 310058, China

[8]School of Energy and Environment, City University of Hong Kong, Hong Kong SAR, China

[9]These authors contributed equally: Mengxiong Wu, Jie Li, Chun-Yu Lai.

[*]Corresponding author email: laichunyu@zju.edu.cn, jianhua.guo@uq.edu.au

Corresponding author address: Level 4 Gehrmann Building (60), The University of Queensland, Brisbane Qld 4072 Australia.

1. Supplementary Text

GC-MS operation

The GC was installed with a J&W HP-PLOT Q PT column (Agilent, USA) using He as the carrier gas at a flow rate of 5.58 mL/min. The GC oven was programmed as follows: (1) samples from the ethane bioreactor: 2 min at 45 ^{0}C, ramp at 10 °C/min to 60 ^{0}C where it was held for 6 min. (2) samples from the butane bioreactor: 45 °C for 2 min, and then heated with a rate of 15 °C/min to 100 °C where it was hold for 7 min. Mass spectra were detected in the electron impact mode at 70 eV. The mass spectrometer was operated in Selected Ion Monitoring (SIM) mode to detect m/z signals at 30 and 32 Da (C_2H_6), 58 and 62 Da (C_4H_{10}), 44 and 45 Da (CO_2), 28, 29 and 30 Da (N_2) with a dwell time of 100 ms for each signal. Data processing was performed using the Chemstation program (Agilent, Unite States).

16S rRNA gene amplicon sequencing

Every 2-3 months, 10 mL of biomass samples were taken from the enrichment bioreactors and pelleted by centrifugation (8,000 g for 10 min). DNA extraction was performed using the FastDNA SPIN for Soil kit (MP Biomedicals, USA) according to the manufacture's protocol. The 16S rRNA gene (V6 to V8 regions) amplicon sequencing was done using the universal primer set 926F (5'-AAACTYAAAKGAATTGACGG-3') and 1392R (5'-ACGGGCGGTGTGTRC-3') on a MiSeq system (Illumina, USA) at the Australian Centre for Ecogenomics (ACE, Brisbane, Australia). QIIME2 was used to process the sequencing results following the ACEPipe (https://acepipe.readthedocs.io/en/latest/).

Short- and long-read metagenomic sequencing

For short-read sequencing, total DNA was extracted using FastDNA SPIN for Soil kit (MP Biomedicals, USA) and quality controlled using Nanodrop spectrophotometer (Thermo Fisher Scientific, Wilmington, DE) and Qubit dsDNA HS Assay Kit. Libraries for short-read sequencing were prepared using Illumina Nextera XT DNA library preparation kit and sequenced on NextSeq 500 (Illumina, USA) platform at ACE.

To obtain Nanopore long reads, total DNA was extracted using Qiagen PowerSoil Pro kit (Qiagen, Germany). Quality of extractions was checked using Qubit 1x dsDNA HS Assay Kit on the Qubit Flex Fluorometer (Thermo Fisher Scientific, Wilmington, DE) and the QIAxcel

DNA High Resolution Kit on the QIAxcel Advanced system (Qiagen, Germany). Libraries were prepared and sequenced on PromethION (Oxford Nanopore Technologies, USA).

Phylogenetic analysis of recovered Symbiobacteriia genomes

Genome tree. Phylogenetic placement of the two recovered Symbiobacteriia genomes in current study was performed with the existing '*Ca.* A. nitratireducens' MAG[1] and available Firmicutes genomes in GTDB r207[2,3] using 120 bacterial-specific conserved marker genes. Briefly, marker genes in genomes were identified using Prodigal 2.6[4] and aligned using HMMER 3.3[5]. Trees were inferred using FastTree 2.1.11[6] with WAG+GMMA models. Bootstrap of the constructed tree was performed using workflow 'bootstrap' from GenomeTreeTk v0.1.6 (https://github.com/dparks1134/GenomeTreeTk) with 100 times nonparametric bootstrapping. The tree was visualized using ARB 6.0.6[7] and further refined using Adobe Illustrator (Adobe, USA).

AssA amino acid tree. The different AssAs in '*Ca.* A. nitroreducens' were aligned with reference AssA, BssA, MasD, PflD, IslA and HpdL protein sequences downloaded from Uniprot database using muscle 3.8.31[8]. We applied trimAI 1.4.1[9] to trim gaps in msa. FastTree 2.1.11[6] was used to infer the phylogenetic tree. Bootstrap value was calculated, and tree was visualized as per the genome tree construction.

Metabolite extraction and detection

For metabolite extractions, enrichment cultures (5 mL) collected from ethane and butane bioreactors were centrifuged at 10,000 rpm for 10 min (4 °C) to harvest the cells. The metabolites were extracted from pelleted cells as described previously[1]. The ethyl and butyl succinate standards (custom synthesized by Best of Chemicals, USA) and cell extracts were then processed and analysed using an ultra-high-sensitivity triple quadrupole GC/MS-TQ8050 system (Shimadzu, Japan). The three most abundant fragmentation ions were chosen to monitor, with Transient 1 used as quantifier and the other two as qualifiers (See Supplementary Table 9).

2. Supplementary Tables

Supplementary Table 1. Nitrogen and electron balances for the enrichment cultures capable of coupling anaerobic ethane/butane oxidation to nitrate reduction in the batch tests.

Nitrogen and electron balance (mmol/L)	C_2H_6/ C_4H_{10} oxidized	NO_3^- and NO_2^- reduced	NH_4^+ generated	N_2-N generated	Maximum electrons available from C_2H_6/C_4H_{10} oxidation	Electrons required for NO_3^- reduction *	Nitrogen balance†	Electron balance‡
	0.94	1.85	0.63	1.44	13.18	12.24	0.90	1.08
Ethane bioreactor	1.15	1.99	1.20	1.21	16.05	15.67	0.82	1.02
	1.02	2.11	1.01	1.12	14.23	13.73	0.99	1.03
	0.49	1.74	0.96	0.73	12.77	11.31	1.03	1.13
Butane bioreactor	0.34	1.43	0.71	0.77	8.84	9.56	0.97	0.92
	0.36	1.71	0.74	0.69	9.34	9.41	1.19	0.99

* Electrons required for NO_3^- reduction = NH_4^+ generated × 8 + N_2-N generated × 5.

† Nitrogen balance = (NO_3^- reduced)/ (NH_4^+ generated + N_2-N generated).

‡ Maximum electrons available from ethane (=C_2H_6 oxidized × 14) or butane (=C_4H_{10} oxidized × 26) oxidation divided by electrons required for NH_4^+ and N_2-N production; theoretically higher than 1.0, due to a fraction of carbon assimilated into biomass cells.

Supplementary Table 2. Sequencing statistics of metagenomics and metatranscriptomics. Key features of the metagenome (Illumina short-read and Nanopore long-read sequencing) and metatranscriptomic datasets generated for the C_2H_6- and C_4H_{10}-fed enrichment cultures. Two samples were taken from the C_2H_6-fed bioreactors for short-read sequencing, thus generating two datasets.

		**C_2H_6-fed cultures**	**C_4H_{10}-fed cultures**
**Metagenomic short read sequencing**	# trimmed reads	99,139,736/102,953,198	116,463,546
	% Reads in metagenomic scaffolds	94.1/94.5	96.5
**Long-read sequencing**	# raw nanopore reads (million)	11.8	28.9
	Maximum read length	310,518	658,543
	N50	4,440	544
**Hybrid assembly**	Assembled metagenome size (Mbp)	474.6	314.6
	N50 (bp)	22,096	100,544
	Maximum scaffold (bp)	6,053,062	4,746,968
	# scaffold	100,732	22,756

**Metatranscriptome**	**Total Illumina reads**	**Trimmed Reads**	**non_rRNA reads**	**mRNA mapped to MAGs**
C2-phase1-RNA	125,039,398	116,358,434	82,846,667	34,950,915
C2-phase2-RNA	212,602,712	194,685,456	137,965,946	71,243,694
C4-phase1-RNA	68474852	64,488,864	40,744,382	25,927,776
C4-phase2-RNA	62856468	59,114,204	37,435,066	23,625,080

Supplementary Table 3. Estimated abundance of each lineage recovered from C_2H_6-fed community. Lineages with abundance over 1% were shown in the table.

Bin ID	C2_ Phase1-DNA (%)	C2_ Phase2-DNA (%)	Classification
C2_01	28.46	26.34	d__Bacteria;p__Patescibacteria;c__4484-211;o__4484-211;f__;g__;s__
**C2_SYM**	**14.38**	**15.55**	**d__Bacteria;p__Firmicutes_E;c__Symbiobacteriia;o__;f__;g__;s__**
C2_02	10.75	11.29	d__Bacteria;p__Armatimonadota;c__Fimbriimonadia;o__Fimbriimonadales;f__Fimbriimonadaceae;g__OLB18;s__OLB18 sp001567425
C2_03	6.77	7.56	d__Bacteria;p__Bacteroidota;c__Ignavibacteria;o__Ignavibacteriales;f__Melioribacteraceae;g__DSXH01;s__
C2_04	3.23	3.63	d__Bacteria;p__Chloroflexota;c__Anaerolineae;o__Promineofilales;f__Promineofilaceae;g__Promineofilum;s__
C2_05	3.83	3.30	d__Bacteria;p__Bacteroidota;c__Bacteroidia;o__Flavobacteriales;f__Vicingaceae;g__BCD5;s__BCD5 sp013112825
C2_06	2.36	2.74	d__Bacteria;p__Proteobacteria;c__Gammaproteobacteria;o__Burkholderiales;f__SG8-39;g__;s__
C2_07	2.46	2.45	d__Bacteria;p__Actinobacteriota;c__Acidimicrobiia;o__ATN3;f__ATN3;g__ATN3;s__
C2_08	2.12	1.94	d__Bacteria;p__Chloroflexota;c__Anaerolineae;o__Anaerolineales;f__EnvOPS12;g__UBA7227;s__UBA7227 sp002473085

C2_09	1.91	1.67	d__Bacteria;p__Patescibacteria;c__Microgenomatia;o__UBA1406;f__GWC2-37-13;g__GWC2-37-13;s__GWC2-37-13 sp002050095
C2_10	1.51	1.48	d__Bacteria;p__Bacteroidota;c__Ignavibacteria;o__Ignavibacteriales;f__Ignavibacteriaceae;g__IGN2;s__IGN2 sp013285405
C2_11	1.19	1.37	d__Bacteria;p__Chloroflexota;c__Anaerolineae;o__Anaerolineales;f__EnvOPS12;g__OLB14;s__
C2_12	1.06	1.28	d__Bacteria;p__Acidobacteriota;c__Vicinamibacteria;o__Vicinamibacterales;f__Fen-181;g__;s__
C2_13	1.26	1.10	d__Bacteria;p__Bacteroidota;c__UBA10030;o__UBA10030;f__;g__;s__

Supplementary Table 4. Estimated abundance of each lineage recovered from C_4H_{10}-fed community. Lineages with abundance over 1% were shown in the table.

Bin ID	C4_DNA (%)	Classification
**C4_SYM**	**16.72**	**d__Bacteria;p__Firmicutes_E;c__Symbiobacteriia;o__;f__;g__;s__**
C4_01	15.46	d__Bacteria;p__Chloroflexota;c__Anaerolineae;o__Promineofilales;f__Promineofilaceae;g__JAAYYY01;s__JAAYYY01 sp012515125
C4_02	15.39	d__Bacteria;p__Planctomycetota;c__Phycisphaerae;o__Phycisphaerales;f__SM1A02;g__CAADGN01;s__CAADGN01 sp900696545
C4_03	11.67	d__Bacteria;p__Chloroflexota;c__Anaerolineae;o__Anaerolineales;f__Anaerolineaceae;g__Bellilinea;s__
C4_04	10.50	d__Bacteria;p__Chloroflexota;c__Anaerolineae;o__Anaerolineales;f__EnvOPS12;g__UBA7227;s__UBA7227 sp002473085

C4_05	6.63	d__Bacteria;p__Proteobacteria;c__Alphaproteobacteria;o__Rhizobiales;f__Rhizobiaceae;g__Aquamicrobium_A;s__
C4_06	3.95	d__Bacteria;p__Chloroflexota;c__Anaerolineae;o__Anaerolineales;f__Anaerolineaceae;g__Bellilinea;s__
C4_07	1.33	d__Bacteria;p__Bacteroidota;c__Ignavibacteria;o__Ignavibacteriales;f__Ignavibacteriaceae;g__Ignavibacterium;s__Ignavibacterium sp900696555
C4_08	1.25	d__Bacteria;p__Proteobacteria;c__Gammaproteobacteria;o__Burkholderiales;f__Burkholderiaceae;g__QOAZ01;s__
C4_09	1.23	d__Bacteria;p__Patescibacteria;c__Microgenomatia;o__UBA1406;f__GWC2-37-13;g__GWC2-37-13;s__GWC2-37-13 sp002050095
C4_10	1.16	d__Bacteria;p__Proteobacteria;c__Alphaproteobacteria;o__Rhizobiales;f__Xanthobacteraceae;g__Palsa-892;s__
C4_11	1.14	d__Bacteria;p__Proteobacteria;c__Gammaproteobacteria;o__Burkholderiales;f__Burkholderiaceae;g__SCN-69-89;s__SCN-69-89 sp001724855

Supplementary Table 5. Calculated expression level of each genome in C_2H_6-fed bioreactor. Genomes with expression level >1% in at least one phase were shown.

Bin ID	C2_Phase1-RNA (%)	C2_Phase2-RNA (%)
C2_SYM	62.58	60.35
C2_02	13.01	9.92
C2_04	3.47	4.29
C2_06	2.51	2.58
C2_11	2.68	3.81
C2_12	2.66	2.94
C2_17	0.84	1.04
C2_26	1.60	1.77

Supplementary Table 6. Calculated expression level of each genome in C_4H_{10}-fed bioreactor. Genomes with expression level >1% in at least one phase were shown.

Bin ID	C4_Phase1-RNA (%)	C4_Phase2-RNA (%)
C4_SYM	84.70	84.27
C4_01	2.89	3.59
C4_02	5.09	3.32
C4_03	1.34	1.23
C4_04	2.45	3.54

Supplementary Table 7. Average amino acid identities (AAI) between different AssAs in '*Ca.* A. nitratireducens' genomes recovered from the ethane- (E), propane- (P) and butane-fed (B) bioreactors. AAI values were calculated using Blastp.

AAI	B_AssA1	B_AssA2	B_AssA3	E_AssA1	E_AssA2	E_AssA3	P_AssA1	P_AssA2	P_AssA3
**B_AssA1**	-	89.66	61.95	100.00	90.96	90.73	100.00	93.10	90.74
**B_AssA2**	89.66	-	91.43	89.66	95.93	97.81	89.66	95.94	97.81
**B_AssA3**	61.95	91.43	-	61.95	96.91	100.00	61.95	96.24	100.00
**E_AssA1**	100.00	89.66	61.95	-	90.96	90.73	100.00	93.10	90.73
**E_AssA2**	90.96	95.93	96.91	90.96	-	96.60	90.96	100.00	96.60
**E_AssA3**	90.73	97.81	100.00	90.73	96.60	-	90.73	96.24	100.00
**P_AssA1**	100.00	89.66	61.95	100.00	90.96	90.73	-	93.10	90.74
**P_AssA2**	93.10	95.94	96.24	93.10	100.00	96.24	93.10	-	96.24
**P_AssA3**	90.74	97.81	100.00	90.73	96.60	100.00	90.74	96.24	-

Supplementary Table 8. Average nucleotide identities (ANI) between different AssAs in '*Ca.* A. nitratireducens' genomes recovered from the ethane- (E), propane- (P) and butane-fed (B) bioreactors. ANI values were calculated using Blastn.

AAI	B_AssA1	B_AssA2	B_AssA3	E_AssA1	E_AssA2	E_AssA3	P_AssA1	P_AssA2	P_AssA3
**B_AssA1**	-	89.31	89.57	99.96	89.85	89.57	99.96	89.78	89.57
**B_AssA2**	89.31	-	98.83	89.27	97.42	98.87	89.27	97.46	98.87
**B_AssA3**	89.57	98.83	-	89.61	96.80	99.96	89.61	96.65	99.96
**E_AssA1**	99.96	89.27	89.61	-	89.89	89.61	100.00	89.74	89.61
**E_AssA2**	89.85	97.43	96.80	89.89	-	96.84	89.89	99.84	96.84
**E_AssA3**	89.57	98.87	99.96	89.61	96.84	-	89.61	96.68	100.00
**P_AssA1**	99.96	89.27	89.61	100.00	89.89	89.61	-	89.74	89.61
**P_AssA2**	89.78	97.47	96.65	89.74	99.84	96.69	89.74	-	96.69
**P_AssA3**	89.57	98.87	99.96	89.61	96.84	100.00	89.61	96.68	-

Supplementary Table 9. Optimized multiple reaction monitoring transitions and retention times for ethylsuccinate and butylsuccinate analysed by GC-MS/MS.

Compound	Retention time (min)	Transient 1 (m/z)	Collision Energy (V)	Transient 2 (m/z)	Collision Energy (V)	Transient 3 (m/z)	Collision Energy (V)
Ethyl succinate	9.940	275.0>73.1	35	217.0>55.1	10	275.0>147.2	10
Butyl succinate	12.245	303.0>147.1	10	147.1>73.1	30	231.0>69	10

3. Supplementary Figures

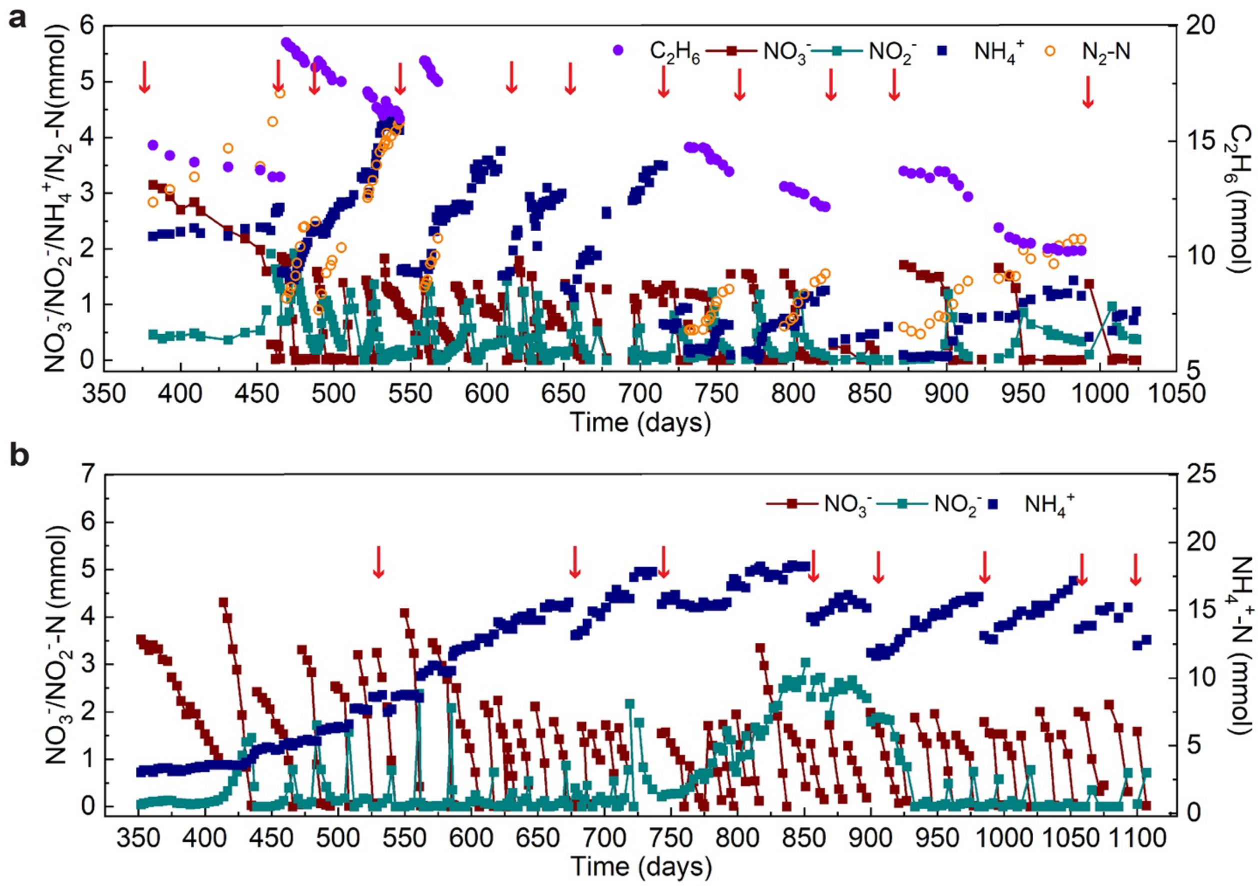

Supplementary Fig. 1 Long-term performance of the C_2H_6- **(a)** and C_4H_{10}-fed **(b)** bioreactors. **(a)** Simultaneous C_2H_6 and NO_3^- consumption with transitory formation of NO_2^-, and production of N_2 and NH_4^+ in the C_2H_6-fed bioreactor. **(b)** NO_3^- consumption with production of NH_4^+ and transitory formation of NO_2^- was also observed in the C_4H_{10}-fed bioreactor. Red arrows indicate medium replacements and the flushing of the bioreactor headspace with C_2H_6 or C_4H_{10}.

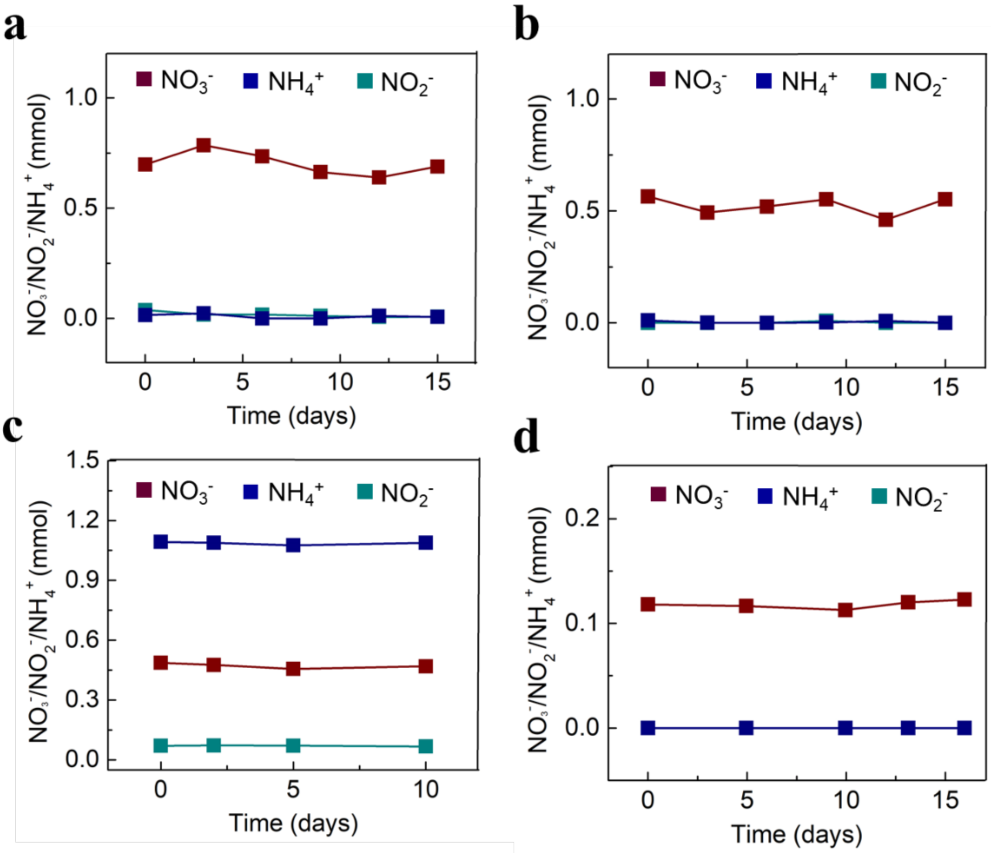

Supplementary Fig. 2 Profiles of NO_3^-, NO_2^- and NH_4^+ in the control tests. a, c, negligible NO_3^- consumption or NO_2^-/NH_4^+ production in the absence of C_2H_6 **(a)** or C_4H_{10} **(c)**. **b, d,** negligible NO_3^- consumption or NO_2^-/NH_4^+ production in the abiotic control (without biomass) supplied with C_2H_6 **(b)** or C_4H_{10} **(d)**.

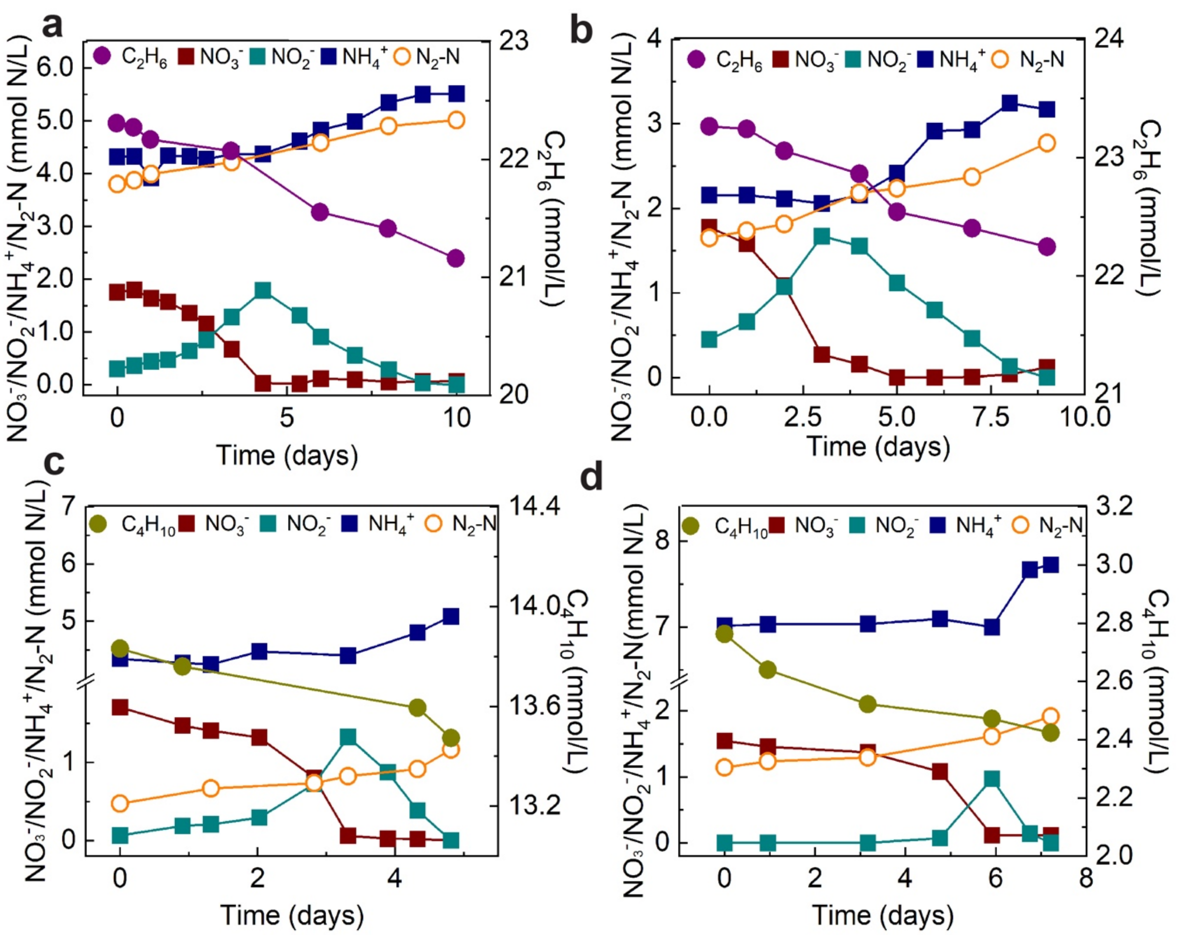

Supplementary Fig. 3 Profiles of C_2H_6, C_4H_{10}, and nitrogen species in the batch tests. a, b, supplementary batch tests for C_2H_6-fed bioreactor (started on Day 522 and 559). **c, d,** supplementary batch tests for C_4H_{10}-fed bioreactor (started on Day 1,100 and 1,124).

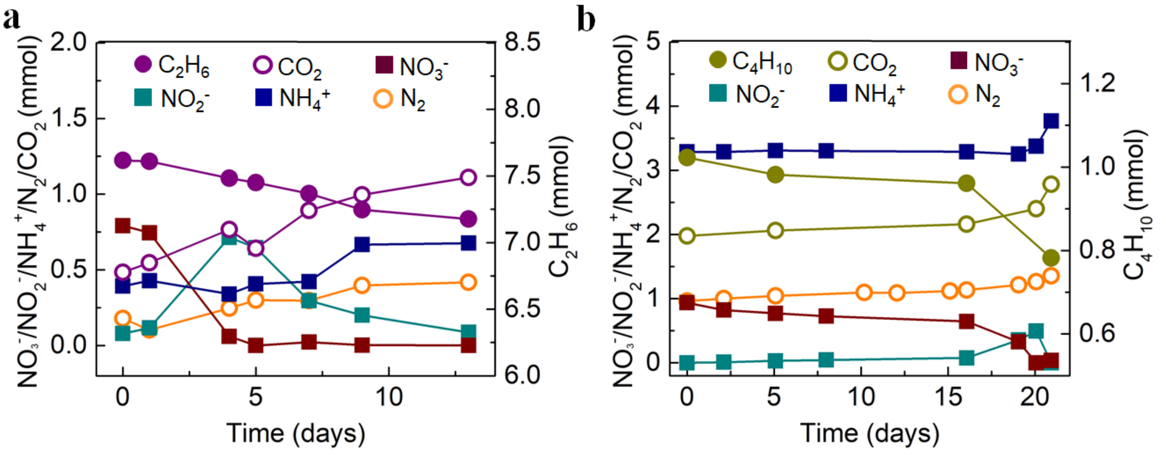

Supplementary Fig. 4 Profiles of total C_2H_6, C_4H_{10}, and nitrogen species during isotope labelling tests. a, b, oxidation of C_2H_6 (**a**) or C_4H_{10} (**b**) to CO_2, and reduction of NO_3^- to NH_4^+ and N_2 with temporary accumulation of NO_2^-.

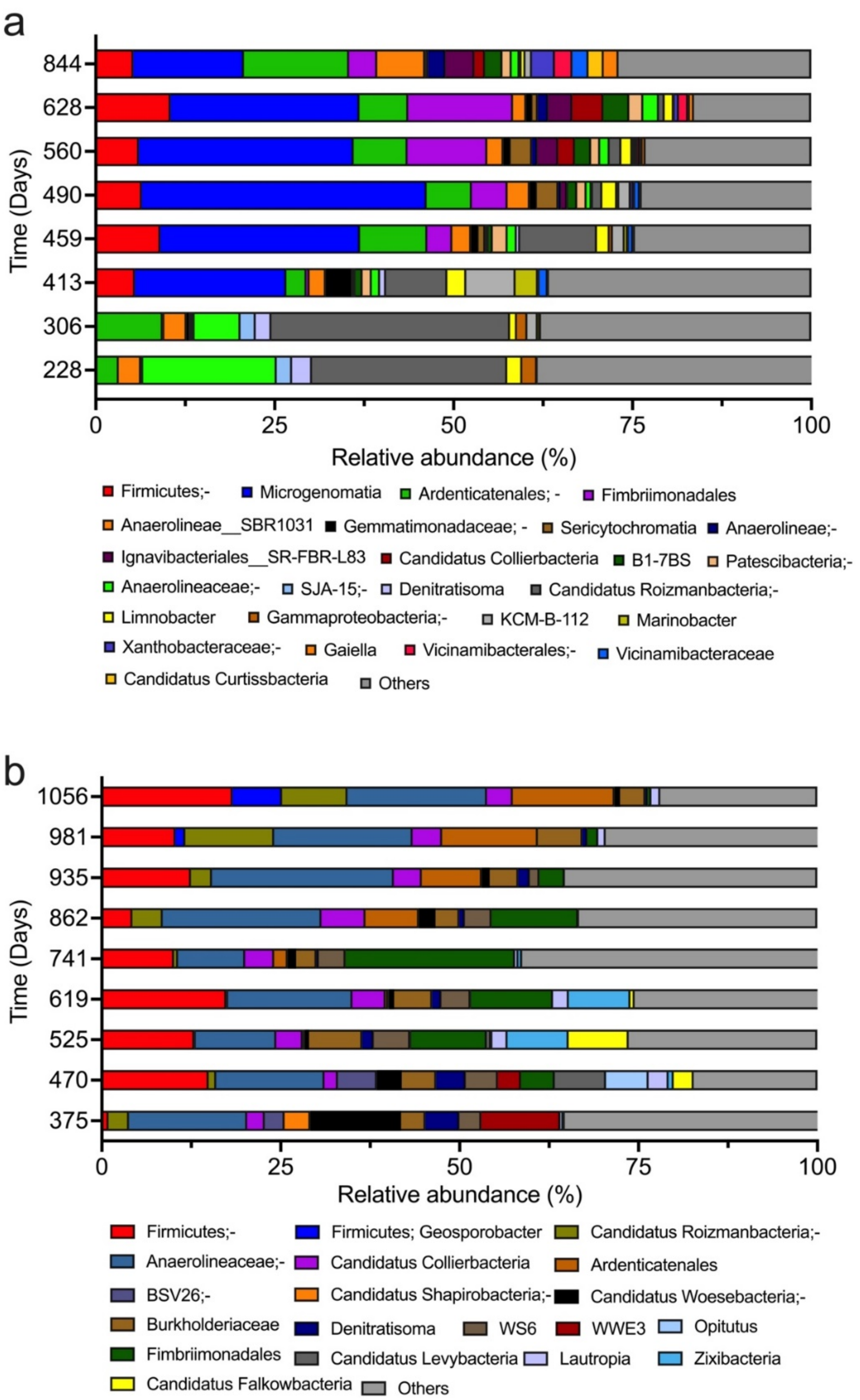

Supplementary Fig. 5 Phylotypes in the enrichment cultures at the genus level based on 16S rRNA gene amplicon sequencing during long-term operations of C_2H_6- **(a)** and C_4H_8-fed **(b)** bioreactors. Genera with an abundance of ≥2% in at least one sample are displayed, while genera account for <2% in all samples are classified as 'Others'.

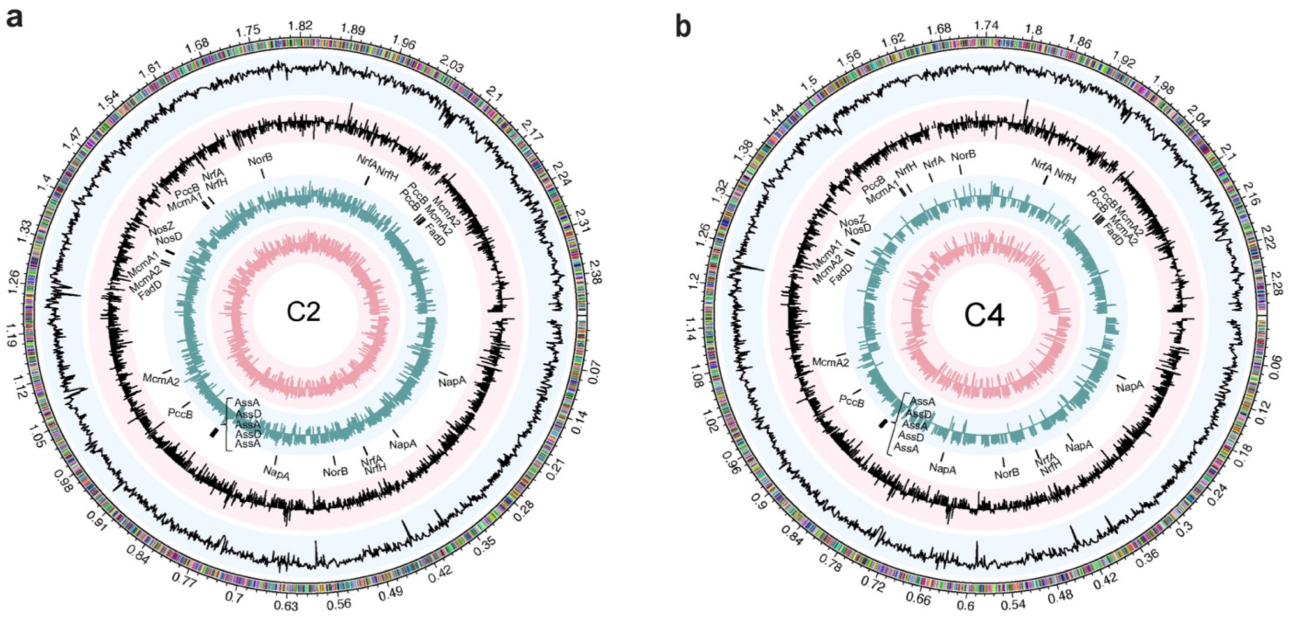

Supplementary Fig. 6 Features of recovered Symbiobacteriia genomes in C_2H_6- (a) and C_4H_8-fed (b) bioreactors. Tracks from outside to inside: Track 1 - the genomes and encoded genes, Track 2- GC ratio, Track 3 - GC skew, Track 4 - loci of genes involved in nitrogen and ethane/butane conversion, Track 5 and 6 - log-transformed gene TPM values in Phase 1 and 2. The lines in Track 5 and 6 facing outward and inward indicate the genes encoded on ‘+’ and ‘-’ strand, respectively. Ass, Alkylsuccinate synthase; Fad, long-chain acyl-CoA synthetase; Mcm, Methylmalonyl-CoA mutase; Pcc, Propionyl-CoA carboxylase; Nap, Nitrate reductase; Nrf, Cytochrome *c* 552 nitrite reductase; Nor, Nitric oxide reductase; Nos, Nitrous oxide reductase.

a

	E_SYM	P_SYM	B_SYM	GCA_003242505	GCA_003242675	GCF_000009905
E_SYM	-	99.96	99.60	55.17	54.38	53.91
P_SYM	99.96	-	99.55	55.23	54.34	53.86
B_SYM	99.60	99.57	-	55.31	54.44	53.95
GCA_003242505	-	-	-	-	61.13	60.33
GCA_003242675	-	-	-	77.45	-	86.49
GCF_000009905	-	-	-	77.20	86.84	-

b

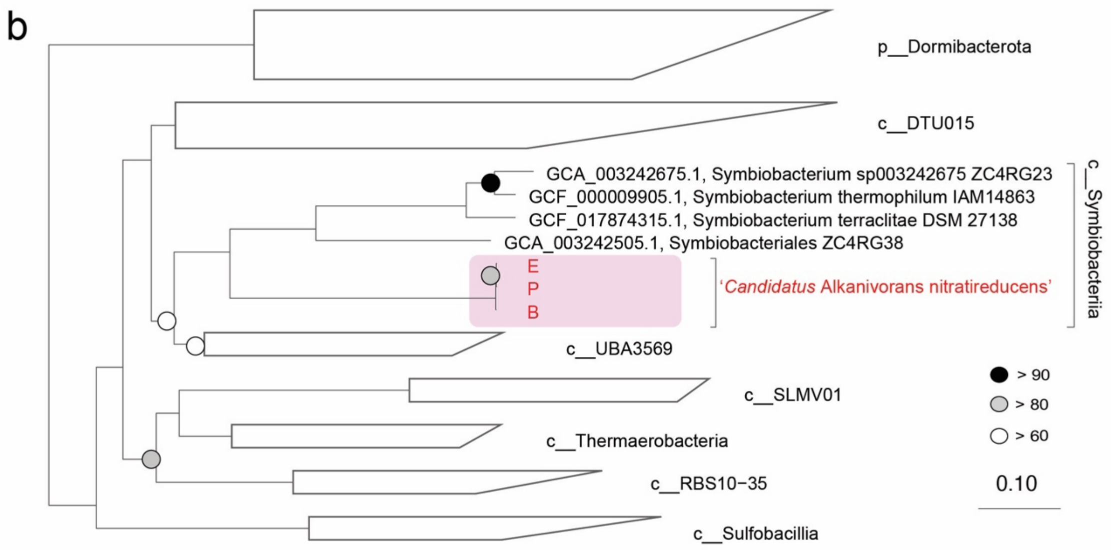

Supplementary Fig. 7 Comparative genome analyses of '*Ca*. A. nitratireducens' in the C_2H_6- and C_4H_{10}-fed systems, and genome-based phylogenetic tree. (a) ANI and AAI between the available Symbiobacteriia genomes. All available genomes including GCA_003242505.1 (*Symbiobacterium*), GCA_003242675 (*Symbiobacterium*) and GCF_000009905 (*S*. thermophilum IAM 14863) were retrieved from GTDB r202. ANI values were calculated using FastANI, filled in lower matrix and scaled in blue, AAI values were calculated using compareM, filled in upper matrix and scaled in red. **(b)** Genome-based phylogenetic tree inferred with a concatenated set of 120 bacterial-specific marker genes. Genomes recovered from C_2H_6-, C_3H_8- and C_4H_{10}-fed bioreactors are highlighted in red text. Genomes from phylum Dormibacterota are used as outgroup.

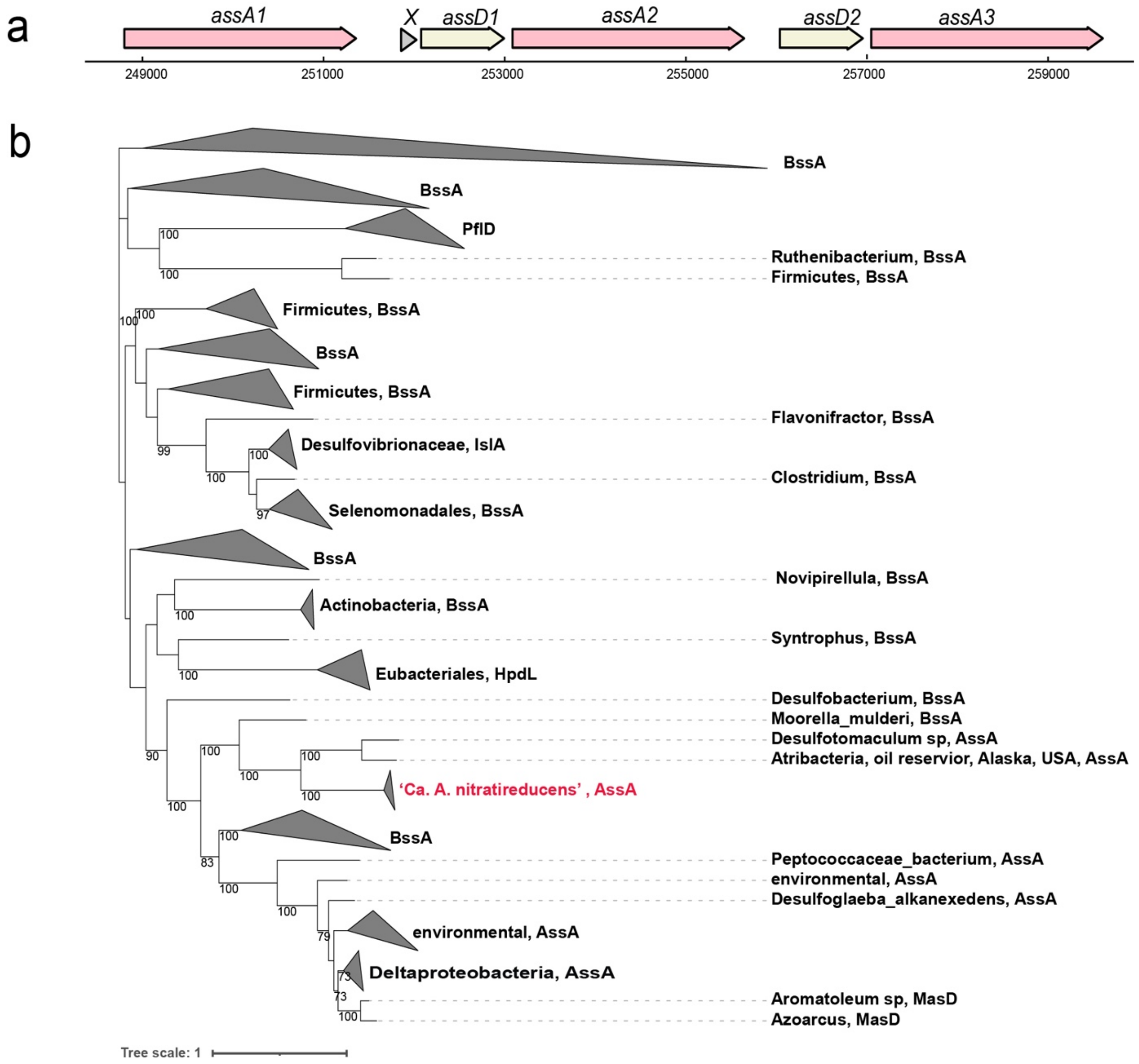

Supplementary Fig. 8 The operon and phylogenetic affiliation of alkylsuccinate synthase (ASS) in '*Ca.* A. nitratireducens'. (a) The ASS operon in '*Ca.* A. nitratireducens' genome recovered from the C_2H_6-fed (E) cultures. Similar with E MAG, the genome of '*Ca.* A. nitratireducens' recovered from the C_4H_{10}-fed cultures also contains three AssA and two AssD subunits. **(b)** Phylogenetic tree of AssA genes. AssA recovered from the three Symbiobacteriia genomes are highlighted in red text. Bootstrap values were determined with 100 non-parametric bootstraps, where 70-100 bootstrap values were shown. The scale bar indicates amino acid substitutions per site.

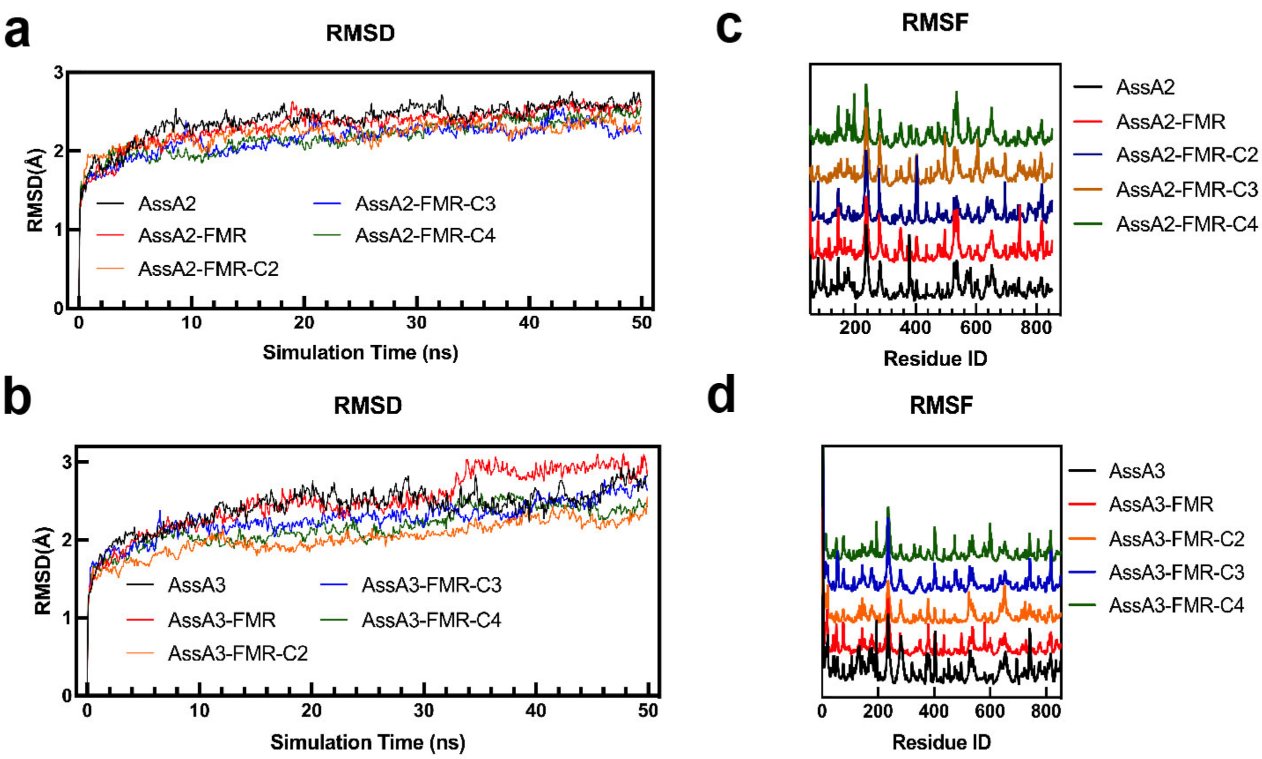

Supplementary Fig. 9 The calculations of root mean square deviation (RMSD) and fluctuation (RMSF) for all molecular dynamics (MD) simulations. a, b, RMSD calculations for MD simulations of AssA2 (**a**) and AssA3 (**b**). **c, d,** RMSF calculations for MD simulations of AssA2 (**c**) and AssA3 (**d**). FMR represents the complex with AssA only binding to fumarate, and FMR-C2/C3/C4 means the AssA binds to both fumarate and ethane/propane/butane.

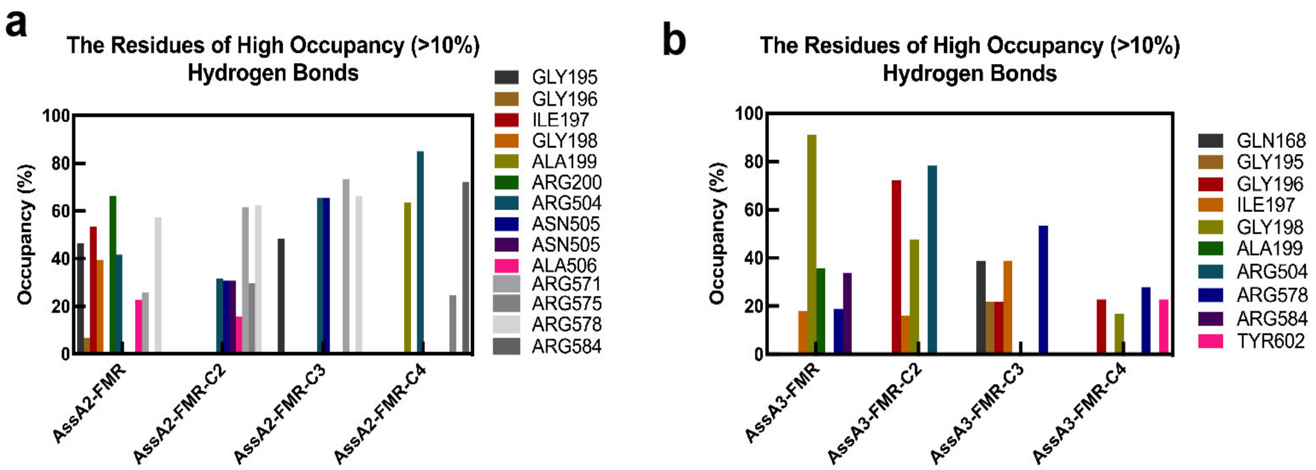

Supplementary Fig. 10 The amino acid residues with occupancy of hydrogen bonds > 10% in AssA2 (a) and AssA3 (b) in '*Ca.* A. nitratireducens'.

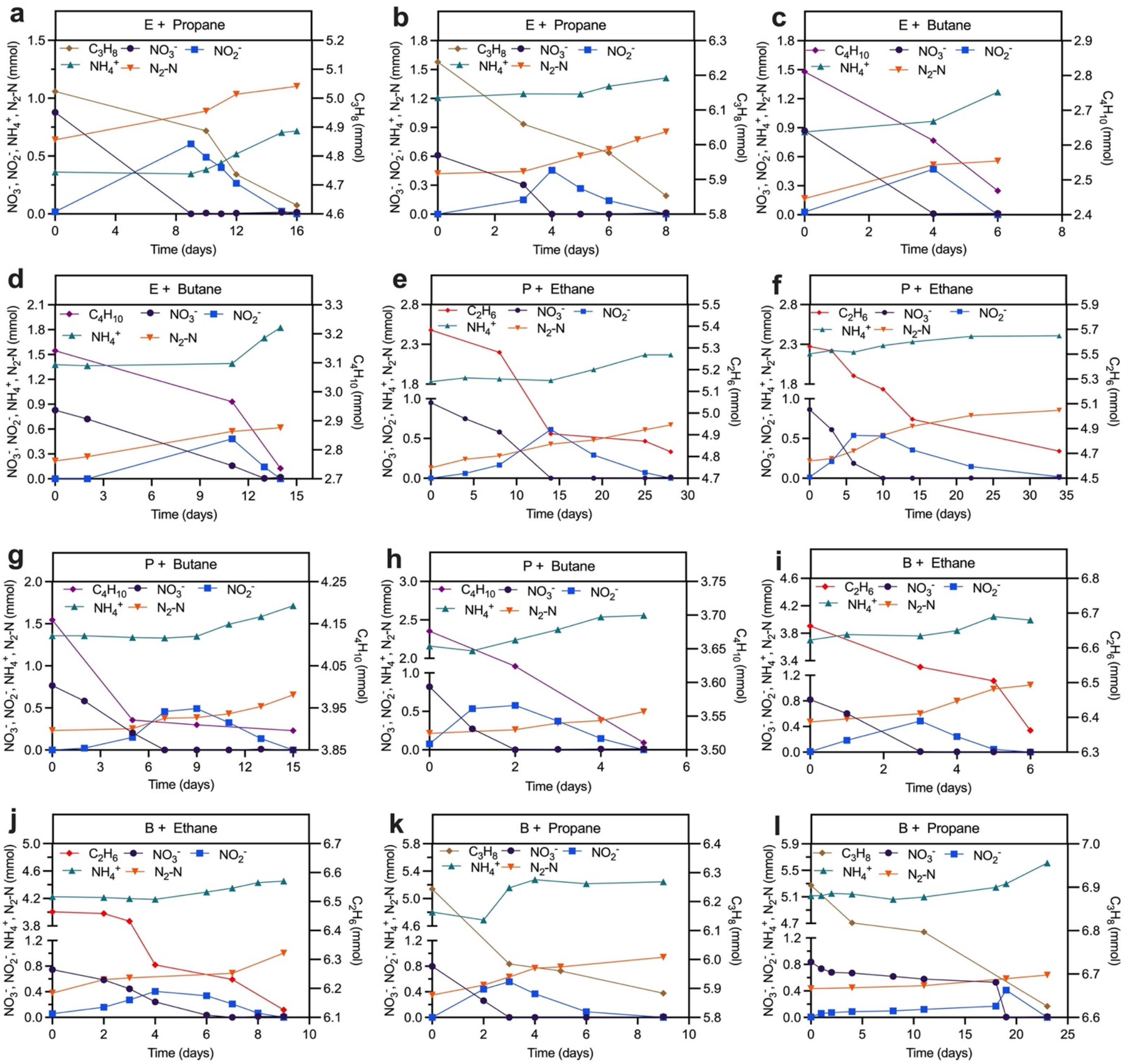

Supplementary Fig. 11 Profiles of C_2H_6, C_3H_8, C_4H_{10}, and nitrogen species in the substrate range tests for '*Ca.* A. nitratireducens'.

1 Wu, M. *et al.* Anaerobic oxidation of propane coupled to nitrate reduction by a lineage within the class Symbiobacteriia. *Nature Communications* **13**, 6115 (2022).

2 Parks, D. H. *et al.* A complete domain-to-species taxonomy for Bacteria and Archaea. *Nat Biotechnol* **38**, 1079-1086 (2020).

3 Parks, D. H. *et al.* A standardized bacterial taxonomy based on genome phylogeny substantially revises the tree of life. *Nat Biotechnol* **36**, 996-1004 (2018).

4 Hyatt, D. *et al.* Prodigal: prokaryotic gene recognition and translation initiation site identification. *BMC Bioinformatics* **11**, 119 (2010).

5 Eddy, S. R. Accelerated profile HMM searches. *PLoS Comput Biol* **7**, e1002195 (2011).

6 Price, M. N., Dehal, P. S. & Arkin, A. P. FastTree 2--approximately maximum-likelihood trees for large alignments. *PLoS One* **5**, e9490 (2010).

7 Ludwig, W. *et al.* ARB: a software environment for sequence data. *Nucleic Acids Res* **32**, 1363-1371 (2004).

8 Edgar, R. C. MUSCLE: multiple sequence alignment with high accuracy and high throughput. *Nucleic acids research* **32**, 1792-1797 (2004).

9 Capella-Gutiérrez, S., Silla-Martínez, J. M. & Gabaldón, T. trimAl: a tool for automated alignment trimming in large-scale phylogenetic analyses. *Bioinformatics* **25**, 1972-1973 (2009).
